# Long non-coding RNA CTBP1-AS2 enhances cervical cancer progression via up-regulation of ZNF217 through sponging miR-3163

**DOI:** 10.1186/s12935-020-01430-5

**Published:** 2020-07-28

**Authors:** Shanshan Yang, Feng Shi, Yuting Du, Zhao Wang, Yue Feng, Jiayu Song, Yunduo Liu, Min Xiao

**Affiliations:** 1 Department of Gynecological Radiotherapy, Harbin Medical University Cancer Hospital, No. 150 Haping Road, Nangang District, Harbin, 150081 Heilongjiang China; 2 Department of Obstetrics and Gynecology, Daqing Longnan Hospital, Daqing, 163453 Heilongjiang China; 3 Department of Gynecological Oncology, Harbin Medical University Cancer Hospital, No. 150 Haping Road, Nangang District, Harbin, 150081 Heilongjiang China; 4 Department of Breast Surgery, Harbin Medical University Cancer Hospital, No. 150 Haping Road, Nangang District, Harbin, 150081 Heilongjiang China

**Keywords:** CTBP1-AS2, miR-3163, ZNF217, Cervical cancer

## Abstract

**Background:**

Long non-coding RNAs (lncRNAs) play significant roles in tumorigenesis and can contribute to identification of novel therapeutic targets for cancers. This paper was aimed at exploring the role of CTBP1 divergent transcript (CTBP1-AS2) in cervical cancer (CC) progression.

**Methods:**

qRT-PCR and western blot assays were used to detect relevant RNA and protein expressions. In vitro functional assays, including CCK8, EdU, TUNEL and transwell assays were applied to explore the functions of CTBP1-AS2 in CC cell proliferation, apoptosis and migration. In vivo animal study was utilized to investigate the role of CTBP1-AS2 in tumor growth. Luciferase reporter, RNA pull down and RIP assays were performed to determine the specific mechanical relationship between CTBP1-AS2, miR-3163 and ZNF217.

**Results:**

CTBP1-AS2 was significantly overexpressed in CC cell lines. Knockdown of CTBP1-AS2 curbed cell proliferation, migration and invasion, while stimulated cell apoptosis in vitro. CTBP1-AS2 facilitated xenograft tumor growth in vivo. Cytoplasmic CTBP1-AS2 was found to be a miR-3163 sponge in CC cells. MiR-3163 inhibition abolished the anti-tumor effects of CTBP1-AS2 knockdown. Additionally, Zinc finger protein 217 (ZNF217) was identified as a direct target of miR-3163. CTBP1-AS2 acted as a miR-3163 sponge to elevate ZNF217 expression. ZNF217 up-regulation abrogated the tumor suppressing effects of CTBP1-AS2 knockdown.

**Conclusion:**

CTBP1-AS2 regulates CC progression via sponging miR-3163 to up-regulate ZNF217.

## Background

Cervical cancer (CC) is the fourth most common diagnosed cancer and the fourth leading cause of cancer-related deaths in females globally [1]. Each year, more than 500,000 cervical cancer cases are diagnosed and approximately 300,000 patients die of cervical cancer worldwide [2]. Human papilloma virus (HPV) is the major cause for the high risk of CC. Based on cancer statistics in 2019, there were 13,170 estimated new cases and 4250 estimated deaths in the United States [3]. Recently, an increasing trend of morbidity and mortality of CC has been discovered in China [4, 5]. Global strategies for the prevention and screening of CC remain to be improved based on various geographic settings and health systems [6]. Preclinical models have been used for the treatment of CC patients [7]. At present, radiotherapy chemotherapy and surgery remain the main clinical therapeutic methods for patients with CC [8–10]. Therefore, it is essential to explore the molecular mechanisms behind the initiation and development of CC.

Long non-coding RNAs (lncRNAs) are a class of RNAs longer than 200 nucleotides but lack the protein-coding potential. Recent findings indicated that lncRNAs play vital roles in gene regulation at the transcriptional level [11]. Dysregulation of lncRNAs is associated with a series of biological processes, such as cell proliferation, apoptosis, invasion and migration [12–14]. Furthermore, lncRNAs have been identified as novel biomarkers of many cancers [15, 16]. To date, the pathologic roles of most lncRNAs remain unknown, which indicates the extensive potential of lncRNAs in the prediction and treatment of various cancers.

In CC, some lncRNAs were discovered to be aberrantly expressed and exerted important biological functions. For instance, linc00511 is highly expressed in CC and knockdown of linc00511 dampens CC cell proliferation and reduces drug resistance to paclitaxel [17]. CTBP1 divergent transcript (CTBP1-AS2), as a newly identified lncRNA, was limitedly reported in cancers. The only report on the role of CTBP1-AS2 in cancer is that CTBP1-AS2 predicts unfavorable prognosis of papillary thyroid cancer [18]. However, the biological role of CTBP1-AS2 in the carcinogenesis and development of CC has not been studied yet.

MicroRNAs (miRNAs) are small ncRNAs with a size between 20 and 25 nt. Based on previous studies, miRNAs can exert various functions in human cancers. For instance, anti-miR-203 suppresses cell growth and stemness in ER-positive breast cancer via targeting SOCS3 [19]. MiR-17-5p and miR-20a-5p inhibit hepatocellular carcinoma metastasis [20]. MiR-17 acts as an oncogene in hepatocellular carcinoma through downregulation of Smad3 [21]. It has been reported that miR-3163 targets ADAM-17 and inhibits the Notch pathway to enhance the sensitivity of HCC cells to antitumor agents in hepatocellular carcinoma [22]. MiR-3163 promotes colorectal cancer cell growth in vivo [23]. This study was aimed to explore the interaction between CTBP1-AS2 and miR-3163.

Zinc finger protein 217 (ZNF217) is protein-coding gene contributing to the tumorigenesis of various human cancers. The coordination between ZNF217 and LSD1 facilitates hepatocellular carcinoma progression [24]. ZNF217 is targeted by miR-211-3p and reverses the effects of miR-211-3p on proliferative and migratory potentials of non-small cell lung cancer cells [25].

To summarize, the current study focused on investigating the role of CTBP1-AS2/miR-3163/ZNF217 axis in the biological behaviors of HPV-positive cells.

## Materials and methods

### Tissue samples

This study was executed between 2014 and 2019, with the ethical approval from the Ethics Committee of Harbin Medical University Cancer Hospital. Patients without Human papillomavirus (HPV) infection were excluded from this study. All 72 participants had signed the written informed consent. Highly sensitive polymerase chain reaction (PCR) techniques were used to detect the HPV. The number of patients infected with HPV-18, HPV-11, HPV-45 and HPV-68 were separately 25, 19, 15, 13. The 72 CC samples and adjacent normal samples from CC patients were collected and instantly maintained in the liquid nitrogen at − 80 °C.

### Cell lines

Human cervical cancer cell lines, including SiHa (HPV positive), HeLa (HPV positive), MS751 (HPV positive) and C33A (HPV negative) as well as the normal cervical epithelial cells (H8) were purchased from Shanghai Institute of Cell Biology (Shanghai, China), cultured routinely in RPMI-1640 medium (Invitrogen, Carlsbad, CA, USA) at 37 °C with 5% CO_2_. 10% fetal bovine serum (FBS; Thermo Fisher Scientific, Waltham, MA, USA) and antibiotics were applied to supplement the culture medium.

### Extraction of total RNA and qRT-PCR

Total RNA was extracted from HeLa and SiHa cells using 1 mL of TRIzol (Invitrogen) and reversely transcribed into cDNA using PrimeScript RT reagent Kit (Takara, Kyoto, Japan) or miRNA reverse transcription PCR kit (Ribobio; Guangzhou, China). The relative gene expression level was measured by SYBR Green PCR Master Mix (Invitrogen) or SYBR^®^ PrimeScript^®^ miRNA RT-PCR Kit (Takara), and Step-One Plus Real-Time PCR System (Applied Biosystems, Foster City, CA, USA), and quantified by the comparative 2^−ΔΔCt^ method. GAPDH mRNA or U6 snRNA served as the endogenous control. The sequences of PCR primers were provided in Additional file 1: Table S1.

### Cell transfection

When the cell density was about 70%, cell transfection was performed in 24-well plates with CO_2_ at 37 °C for 48 h utilizing Lipofectamine 2000 (Invitrogen). The duplicate short hairpin RNAs for CTBP1-AS2, ZNF217 (sh-CTBP1-AS2#1/2, sh-ZNF217#1/2), plasmid pcDNA3.1/ZNF217 were designed by Genepharm (Shanghai, China), as well as their relative negative control RNA (sh-Ctrl, pcDNA3.1). MiR-3163 mimics and NC mimics, miR-3163 inhibitor and NC inhibitor were also produced by Genepharm. Relevant sequences were provided in Additional file 1: Table S1.

### Cell counting kit-8 (CCK-8)

HeLa or SiHa cells were planted into 96-well plates at 3 × 10^3^ per well. After incubated with different time points, cell viability was evaluated by adding 10 μl of CCK-8 solution (Beyotime, Shanghai, China) for 2 h following suppliers guide. The proliferation activity (OD value) was detected at 450 nm by microplate reader (Bio-Rad, Hercules, CA, USA).

### EdU staining

EdU assay kit from Ribobio (Guangzhou, China) was added into cell culture medium in 96-well plates for 3 h. Then, 5 × 10^4^ cells were subjected to 4% paraformaldehyde fixation, 0.5% Troxin X-100 incubation and 1 × Apollo^®^ 488 fluorescent staining. Cell nucleus was subjected to DAPI staining in the dark, and observation using microscope (Thermo Fisher Scientific).

### TUNEL staining

TUNEL staining was used to detect cell apoptosis following the guidelines of in situ Cell Death Detection Kit (Roche Diagnostics GmbH, Penzberg, Germany). Transfected cells (1 × 10^5^) were washed in PBS and stained by TUNEL kit. After treatment with DAPI solution, positively stained cells were all counted using EVOS FL microscope (Thermo Fisher Scientific).

### Transwell assays

The transwell chamber (Corning Incorporated, Corning, NY, USA) coated with Matrigel (BD Biosciences, Franklin Lakes, NJ) at high concentration or not was employed for cell invasion or migration assay. HeLa and SiHa cells (1 × 10^5^) were added into the upper chamber with serum-free medium. Conditioned culture medium was put into the lower chamber. The invaded or migrated cells were treated with 4% paraformaldehyde fixation and crystal violet solution after 48 h, followed by counting under the microscope (Thermo Fisher Scientific).

### Western blotting

Protein samples of 5 × 10^5^ cells were prepared in RIPA lysis buffer (Beyotime) on ice and quantified. 50 μg of samples were subjected to 10% SDS PAGE separation, then transferred to PVDF membranes (Millipore, Billerica, MA, USA). Following sealing with 5% skimmed milk for 1 h, membranes were incubated with primary antibodies including anti-Bax (ab32503), anti-Bcl-2 (ab196495), anti-caspase 3 (ab13847), anti-cleaved caspase-3 (ab2302), anti-ZNF217 (ab136678) and anti-GAPDH (ab128915), together with corresponding HRP-tagged secondary antibodies (all from Abcam, Cambridge, MA, USA). GAPDH served as internal control. Samples were analyzed by enhanced chemiluminescence reagent (Santa Cruz Biotechnology, Santa Cruz, CA, USA).

### Subcellular fractionation assay

The separation of nucleus and cytoplasm was run in HeLa and SiHa cells (1 × 10^7^) with PARIS Kit (Invitrogen) on the basis of protocol. After centrifugation, cells were treated with cell fractionation buffer to isolate cell cytoplasm. Cell nucleus was acquired via adding cell disruption buffer. GAPDH and U6 acted as the fractionation indicators for cell cytoplasm and cell nucleus, respectively. Quantification of CTBP1-AS2, GAPDH and U6 in different cellular fractions was made by qRT-PCR.

### Fluorescence in situ hybridization (FISH) assay

The RNA FISH probe for CTBP1-AS2 was bought from RiboBio and utilized as suppliers requested. Cells were cultivated with FISH probe in hybridization buffer. Cell nuclei were then subjected to Hoechst counterstaining, finally imaged by laser scanning confocal microscope from ZEISS (Jena, Germany).

### Dual-luciferase reporter gene assays

The wild type (WT) dual-luciferase reporter gene vectors pmirGLO-CTBP1-AS2 WT and pmirGLO-ZNF217 WT were obtained using the predicted miR-3163 binding sites to CTBP1-AS2 sequence or 3′ un-translated region (3′UTR) of ZNF217. The mutant (MUT) vectors pmirGLO-CTBP1-AS2 MUT and pmirGLO-ZNF217 MUT were established with point mutations of miR-3163 binding sites. Relevant sequences were provided in Additional file 1: Table S1. All vectors were co-transfected into cells with miR-3163 mimics or NC mimics for 48 h. The pmirGLO dual-luciferase reporter vectors were bought from Promega (Madison, WI). Dual-Luciferase Reporter Assay System (Promega, Madison, WI, USA) was applied for detecting vector activity.

### RNA pull-down assay

RNA pull down assay was undertaken using Pierce Magnetic RNA–Protein Pull-Down Kit (Thermo Fisher Scientific, Waltham, MA, USA). The wild type or mutant CTBP1-AS2 sequences containing the putative miR-3163 binding sites were labeled with the Biotin. Cell lysates of 1 × 10^6^ cells were mixed with Biotin labeled CTBP1-AS2 for 1 h, then with streptavidin beads for 30 min. The enrichment of miR-3163 was analyzed by qRT-PCR.

### RNA immunoprecipitation (RIP)

Using EZMagna RIP Kit (Millipore), RIP assay was conducted in HeLa and SiHa cells (1 × 10^7^). Lysates from RIP lysis buffer were subjected RIP buffer incubation with anti-Ago2 or anti-IgG antibodies-coated beads (Millipore) for 4 h. At length, the precipitated RNAs were isolated and purified, result was analyzed by RT-qPCR.

### Animal study

BALB/c female nude mice from Shanghai SIPPR-BK Laboratory Animal (Shanghai, China) were used for in vivo experiment and maintained under SPF-condition lab. The animal-related protocol was approved by the Animal Research Ethics Committee of Harbin Medical University Cancer Hospital. SiHa cells stably transfected with sh-Ctrl and sh-CTBP1-AS2#1 were injected into nude mice at a density of 5 × 10^6^. The tumor volumes were recorded every 4 days and calculated in accordance with a formula (length × width^2^ × 0.5). Twenty-eight days later, tumors were excised from killed mice and weighed for further analysis.

### Statistical analysis

Prism 6 software (GraphPad, San Diego, CA, USA) was utilized for analyzing all data from three independent replications. Data were exhibited as mean ± SD. Group difference was compared by Student’s t test or one-way/two-way ANOVA, and data were considered significant when p < 0.05. Kaplan–Meier survival analysis was conducted to analyze the significance of high or low expression of CTBP1-AS2/miR-3163**/**ZNF217 in overall survival of CC patients.

## Results

### CTBP1-AS2 is aberrantly up-regulated in CC cells and silenced CTBP1-AS2 curbs the malignant behaviors of CC cells

**Fig. 1:**
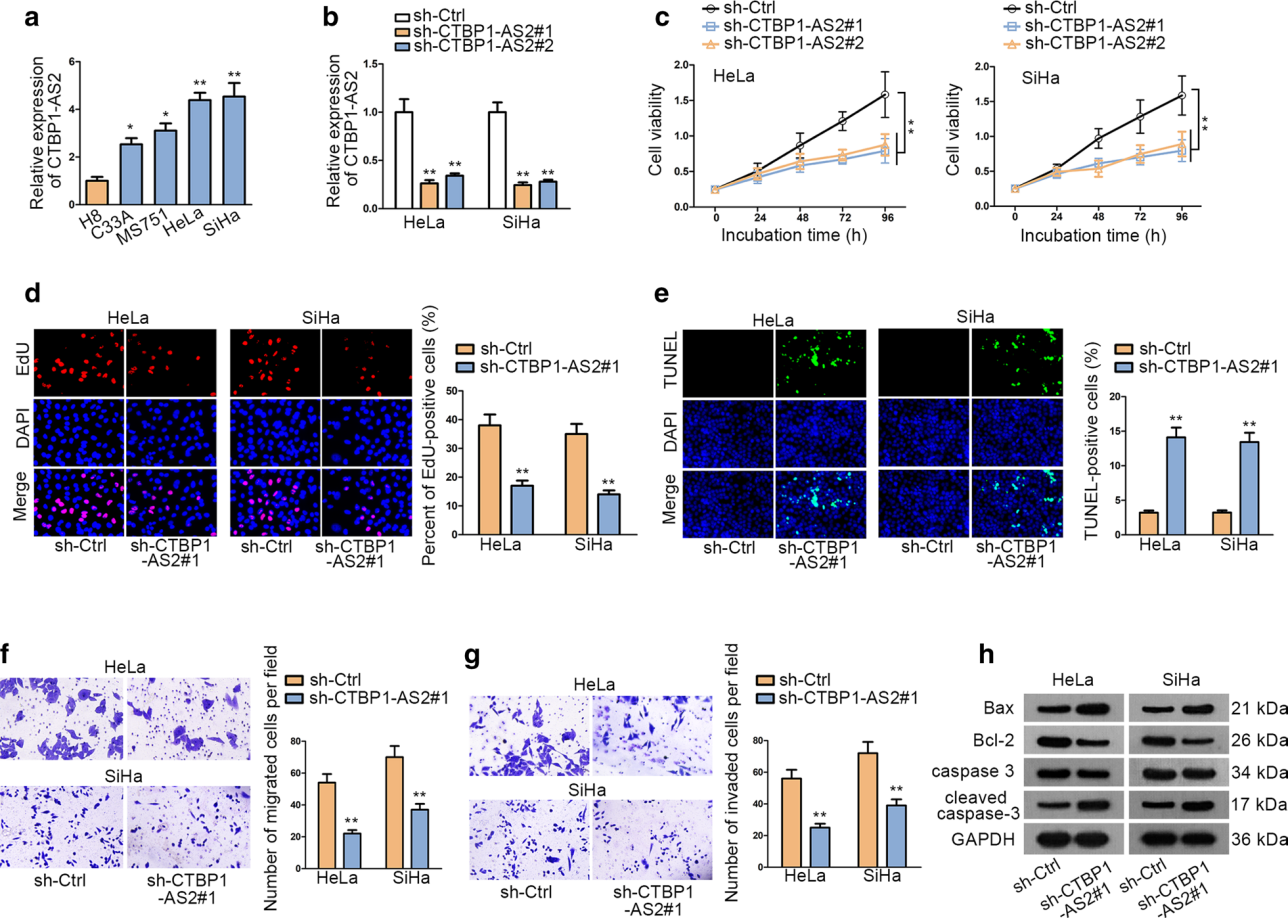
CTBP1-AS2 is aberrantly up-regulated in CC cells. **a** qRT-PCR assay was used to examine CTBP1-AS2 expression in CC cell lines and normal cell line. **b** The transfection efficiency of sh-CTBP1-AS2#1/2 plasmid was guaranteed by qRT-PCR. **c**, **d** CCK-8 and EdU were performed to detect cell proliferation. **e** TUNEL was used to examine apoptosis. **f**, **g** Transwell assays were conducted to measure cell migration and invasion ability respectively. **h** Western blot was performed to examine the expression of apoptosis-related proteins. ^*^P < 0.05, ^**^P < 0.01

At first, we performed qRT-PCR to explore the expression of CTBP1-AS2 in CC cell lines (C33A, MS751, HeLa and SiHa). CTBP1-AS2 was found abnormally up-regulated in CC cell lines than in normal cervical epithelial cell line (H8) (Fig. 1a). The highest level of CTBP1-AS2 was detected in SiHa and HeLa cells (HPV-positive). Then, we performed loss-of-function experiments in Hela and SiHa cell lines after guaranteeing the transfection efficiency of sh-CTBP1-AS2#1/2 plasmid by qRT-PCR (Fig. 1b). Then, CCK8 and EdU assays were performed to determine the effect of CTBP1-AS2 on cell proliferation. We observed that knockdown of CTBP1-AS2 significantly suppressed cell proliferation (Fig. 1c, d). TUNEL assay was performed to investigate cell apoptosis. We found significantly enhanced apoptosis ability after silencing CTBP1-AS2 (Fig. 1e). Results of transwell assays indicated that knockdown of CTBP1-AS2 notably reduced the number of migrated and invaded cells (Fig. 1f, g). As for cell apoptosis, we found that knockdown of CTBP1-AS2 significantly increased the expression of Bax and cleaved caspase-3, while decreased the expression of Bcl-2 was observed through western blot assay (Fig. 1h). To further prove the oncogenic role of CTBP1-AS2 in CC, we also conducted gain-of function assays in normal H8 cells. After overexpression of CTBP1-AS2 in H8 cells (Additional file 2: Figure S1A), cell proliferation was strengthened, whereas apoptosis rate was decreased (Additional file 2: Figure S1B–D). In addition, the migration and invasion were both stimulated by the overexpression of CTBP1-AS2 (Additional file 2: Figure S1E–F). Taken together, CTBP1-AS2 exerts positive effects on CC cell growth, migration and invasion in vitro.

### CTBP1-AS2 acts as a miR-3163 sponge in CC cells

**Fig. 2:**
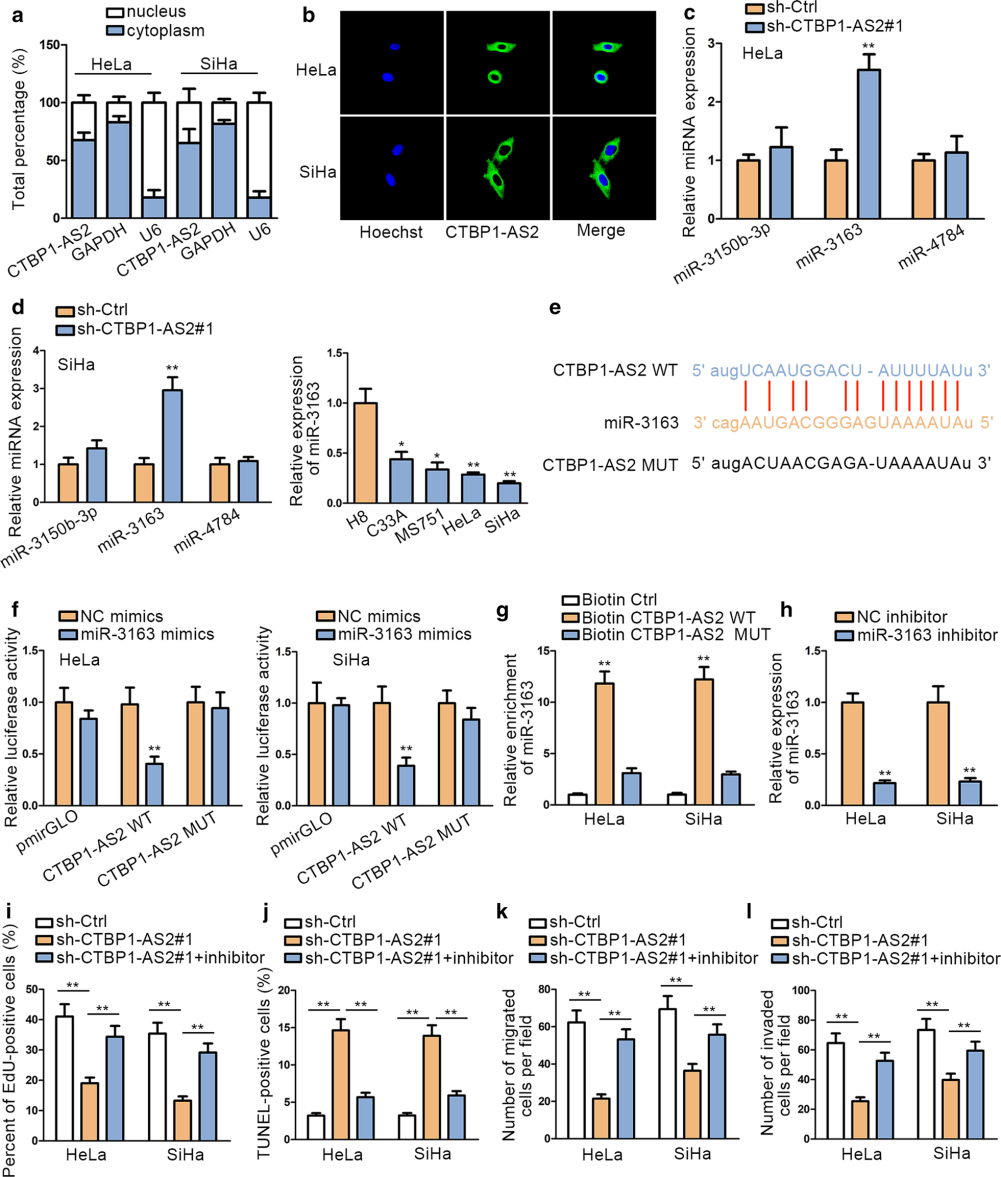
CTBP1-AS2 acts as a miR-3163 sponge in CC. **a**, **b** Subcellular fractionation and FISH assays were performed to determine the subcellular location of CTBP1-AS2. **c** qRT-PCR analysis was performed to evaluate miR-3163 expression after knockdown of CTBP1-AS2. **d** qRT-PCR analysis was conducted to examine the expression of miR-3163 in CC cell lines and normal cell lines. **e** Putative binding of miR-3163 and CTBP1-AS2 was predicted by starBase tool. **f** Dual luciferase reporter assays were performed in HeLa and SiHa cell lines. **g** RNA pull down was performed to determine the interaction between miR-3163 and CTBP1-AS2. **h** The transfection efficiency of miR-3163 inhibitor was determined by qRT-PCR. **i** EdU was performed to determine cell proliferation with inhibited miR-3163 to rescue down-regulated CTBP1-AS2. **j** TUNEL was conducted to examine cell apoptosis with inhibited miR-3163 to rescue down-regulated CTBP1-AS2. **k**, **l** Transwell assays were used to measure cell migration and invasion with inhibited miR-3163 to rescue CTBP1-AS2. ^**^P < 0.01

It has been widely reported that lncRNAs exert different functions in nucleus and cytoplasm at a transcription or post-transcription level. In order to determine the potential role of CTBP1-AS2 in CC cells, we detected its subcellular location. Subcellular fractionation manifested that CTBP1-AS2 was principally located in the cytoplasm of CC cells (Fig. 2a). The result of FISH assay further confirmed this conclusion, demonstrating the post-transcriptionally regulatory role of CTBP1-AS2 (Fig. 2b). We detected combinable miRNAs for CTBP1-AS2 via Starbase database (http://starbase.sysu.edu.cn/) and restricted binding conditions (medium stringency >=2 in Clip data, with or without data in degradome data, class >=7 mer-m8 and Ago ExpNum >=5). Three miRNAs (miR-3150b-3p, miR-3163 and miR-4784) were identified. qRT-PCR was performed to measure miRNA expression after silencing CTBP1-AS2. The expression of miR-3163 exhibited the most significant up-regulation after knockdown of CTBP1-AS2 compared with negative control group (Fig. 2c). Besides, miR-3163 has been reported to be a tumor suppressor in Retinoblastoma Cancer Stem Cells (RCSCs) [26]. Therefore, we selected miR-3163 as candidate miRNA for this study. In the present study, we noticed a significant down-regulation of miR-3163 in CC cell lines, which is contrary to the expression profile of CTBP1-AS2 (Fig. 2d). We obtained the putative miR-3163 binding site in the sequence of CTBP1-AS2 by utilizing Starbase (Fig. 2e). Next, dual luciferase reporter assays were performed in HeLa and SiHa cells to determine the physical interaction between CTBP1-AS2 and miR-3163. We observed that transfection of miR-3163 mimics evidently attenuated the luciferase activity of CTBP1-AS2-WT, but not CTBP1-AS2-MUT (Fig. 2f). RNA pull down assay manifested that miR-3163 was significantly enriched in biotinylated CTBP1-AS2-WT compared to negative control groups, while no products were observed in biotinylated CTBP1-AS2-MUT (Fig. 2g), which validated the combination between CTBP1-AS2 and miR-3163. From above results, we verified that miR-3163 can bind with CTBP1-AS2.

To determine the effects of miR-3163 on CTBP1-AS2-mediated biological functions in CC cells, we knocked down the expression of miR-3163 (Fig. 2h) and performed rescue experiments in CC cells. EdU assay revealed that miR-3163 inhibition could abrogate the anti-proliferation effect of sh-CTBP1-AS2 (Fig. 2i). Results of TUNEL assay demonstrated that miR-3163 inhibition could reverse the pro-apoptosis effect of sh-CTBP1-AS2 (Fig. 2j). Data of transwell assays manifested that miR-3163 inhibition could reverse the inhibitory effects of silencing CTBP1-AS2 on migration and invasion (Fig. 2k, l). Based on these results, we identified miR-3163 as a tumor suppressor in CC.

### MiR-3163 targets ZNF217 in CC cells

**Fig. 3:**
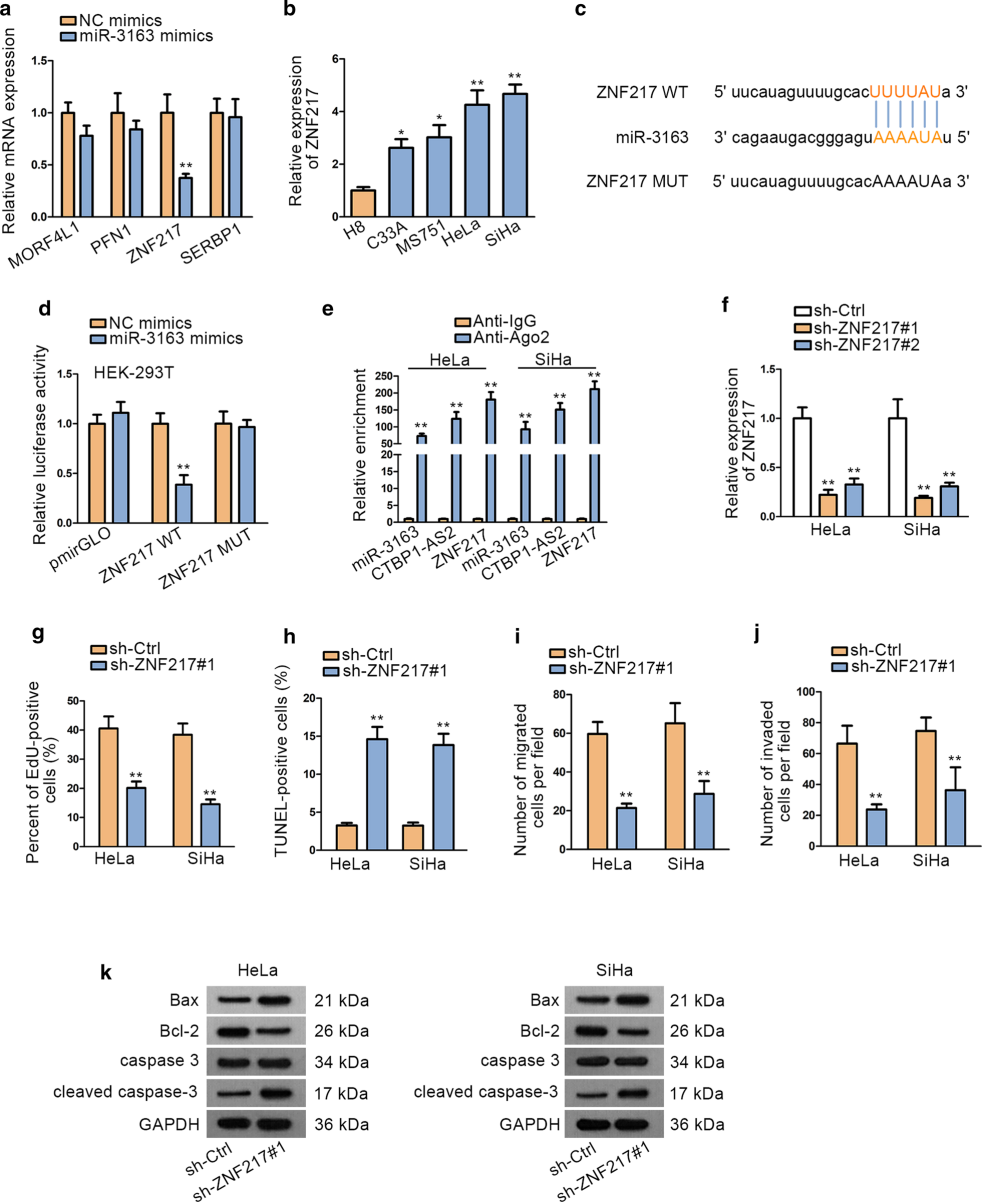
MiR-3163 targets ZNF217 in CC. **a** qRT-PCR was performed to study the expression of target genes after overexpressing miR-3163. **b** qRT-PCR analysis was conducted to examine the ZNF217 expression in CC cell lines and normal cell lines. **c** Potential binding sites between ZNF217 and miR-3163 were predicted by starBase website. **d** Luciferase reporter assay was performed to detect the interaction between ZNF217 and miR-3163 in HEK-293T. **e** RIP demonstrated that miR-3163, CTBP1-AS2 and ZNF217 co-existed in RISC. **f** The transfection efficiency of sh-ZNF217 plasmids was ensured by qRT-PCR. **g**–**k**. Functional experiments was carried to study the role of ZNF217 in CC cells. ^*^P < 0.05, ^**^P < 0.01

Numerous miRNAs have been discovered to be dysregulated in various cancers and exert their functions through modulating their downstream target genes. We found four target genes (MORF4L1, PFN1, ZNF217 and SERBP1) of miR-3163 using Starbase database (high stringency >=3 in Clip data, with or without data in degradome data, microT program, and Ago ExpNum >=35). qRT-PCR was performed to detect the effects of overexpressing miR-3163 on expression of 4 mRNAs. We observed that the expression of ZNF217 was notably down-regulated after overexpressing miR-3163 while the other three mRNAs were not influenced (Fig. 3a). Besides, ZNF217 was discovered to be an oncogene in several cancers. For example, ZNF217 expression is predominantly increased in prostate cancer (PCa) and promotes PCa growth [27]. ZNF217 has been found to be an indicator of bone metastasis in breast cancer [28]. In the present study, we found that ZNF217 was also significantly highly expressed in CC cell lines (Fig. 3b), which was contrary to the expression status of miR-3163 in CC cell lines. We obtained putative miR-3163 binding site in 3′ UTR sequence of ZNF217 from starBase (Fig. 3c). Luciferase reporter assay performed in HEK-293T manifested that miR-3163 mimics could weaken the luciferase activity of ZNF217-WT (Fig. 3d). RIP was performed to verify the interaction among CTBP1-AS2, miR-3163 and ZNF217. The results revealed the significant enrichment of miR-3163, ZNF217 and CTBP1-AS2 in anti-Ago2 group compared with IgG control group (Fig. 3e). To determine the role of ZNF217 in CC cells, we conducted loss-of-function experiments in HeLa and SiHa cells. qRT-PCR analysis determined the transfection efficiency of sh-ZNF217 firstly (Fig. 3f). According to the results of EdU assay, knockdown of ZNF217 inhibited cell proliferation (Fig. 3g). TUNEL results showed that silencing ZNF217 promoted CC cell apoptosis ability (Fig. 3h). Silencing ZNF217 also curbed CC cell migration and invasion, as shown on Fig. 3i and j. Western blot was performed to measure the expression level of apoptosis-related proteins. Consist with the result of TUNEL assay, ZNF217 knockdown promoted the apoptosis of CC cells (Fig. 3k). Collectively, these functional assays revealed that ZNF217 acts as an oncogene in CC progression through facilitating CC cell proliferation, invasion, migration and inhibiting CC cell apoptosis.

### ZNF217 overexpression reverses the anti-tumor effects of CTBP1-AS2 knockdown

**Fig. 4:**
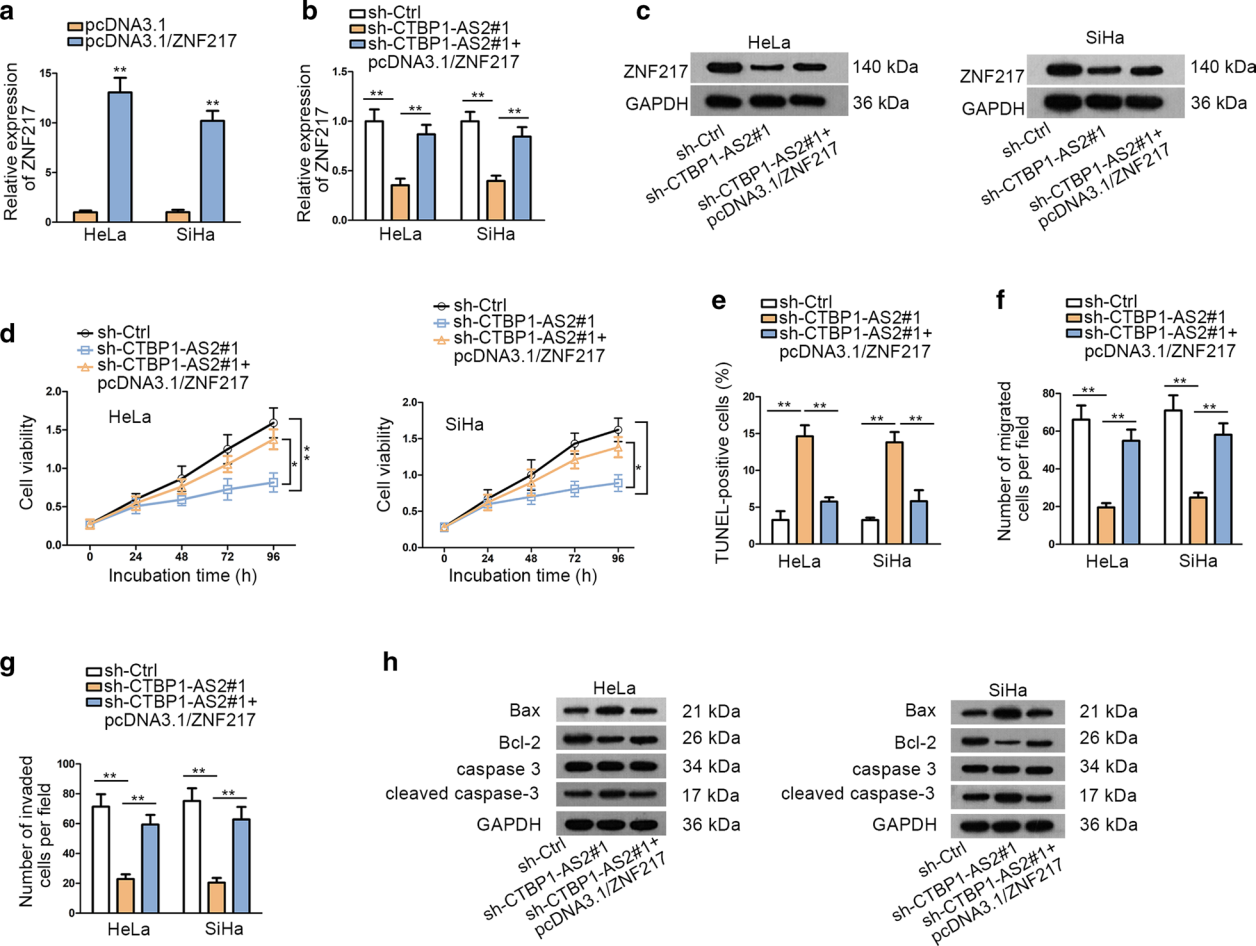
ZNF217 overexpression reverses the anti-tumor effects of CTBP1-AS2 knockdown. **a** qRT-PCR analysis was used to determine the expression of ZNF217 after transfecting with pcDNA3.1 and pcDNA3.1/ZNF217 plasmids. **b**, **c** qRT-PCR and western blot were performed to detect the expression of ZNF217 mRNA and protein. **d** CCK8 assay was used to evaluate cell vitality. **e** TUNEL assay was conducted to evaluate cell apoptosis. **f**, **g** Transwell assays were performed to measure cell migration and invasion ability respectively. **h** Western blot was performed to measure the expression of apoptosis-related proteins. ^*^P < 0.05, ^**^P < 0.01

To further investigate the CTBP1-AS2/miR-3163/ZNF217 axis in CC, we performed rescue assays. Firstly, we performed qRT-PCR analysis to identify the transfection efficiency of pcDNA3.1/ZNF217 (Fig. 4a). qRT-PCR and western blot analysis were conducted to study the expression of ZNF217 after knockdown of CTBP1-AS2. The results showed that the mRNA and protein levels of ZNF217 were inhibited significantly after knockdown of CTBP1-AS2, but increased again when co-transfected with pcDNA3.1/ZNF217 (Fig. 4b, c). Subsequently, a series of rescue experiments was performed to study the influence of up-regulated ZNF217 on CTBP1-AS2-induced cellular function. CCK8 showed that cell vitality was suppressed after silencing CTBP1-AS2, but enhanced again after ZNF217 overexpression (Fig. 4d). Cell cycle distribution was also analyzed by flow cytometry analysis. Cell cycle was arrested at G0/G1 phase by the silencing of CTBP1-AS2, whereas this tendency was reversed by the upregulation of ZNF217 (Additional file 3: Figure S2A). The proliferation marker PCNA and cell cycle related proteins (CDK1 and Cyclin D1) were also detected in two CC cells transected with sh-Ctrl, sh-CTBP1-AS2#1 or co-transfected with sh-CTBP1-AS2#1 and pcDNA3.1/ZNF217. As expected, all the levels of above proteins were reduced by the downregulation of CTBP1-AS2 but was recovered by the overexpression of ZNF217 (Additional file 3: Figure S2B). Results of TUNEL assay manifested that the apoptotic cells increased significantly after knockdown of CTBP1-AS2, but decreased again after transfecting pcDNA3.1/ZNF217 (Fig. 4e). Results of transwell revealed that ZNF217 overexpression abrogated the anti-migration and anti-invasion effects of CTBP1-AS2 knockdown in vitro (Fig. 4f, g). In addition, western blot assay further validated that ZNF217 overexpression could reverse the pro-apoptosis effects of sh-CTBP1-AS2 (Fig. 4h). These results indicated that ZNF217 overexpression could rescue the oncogenic function of CTBP1-AS2.

### CTBP1-AS2 activates PI3K/AKT and Src/FAK signaling pathways in CC cells by upregulating ZNF217

PI3K/AKT pathway can involve in cell proliferation and apoptosis. Moreover, Src/FAK pathway is known as a regulator for cell migration and invasion. Here, we detected whether CTBP1-AS2 regulated CC cell growth and migration through these two signaling pathway. Through western blot analyses, the phosphorylated levels of PI3K, AKT, mTOR, Src and FAK were all reduced by the inhibition of CTBP1-AS2, while were enhanced again by the enhancement of ZNF217 expression (Additional file 3: Figure S2C-D).

### CTBP1-AS2/miR-3163/ZNF217 axis accelerates in vivo tumor growth

**Fig. 5:**
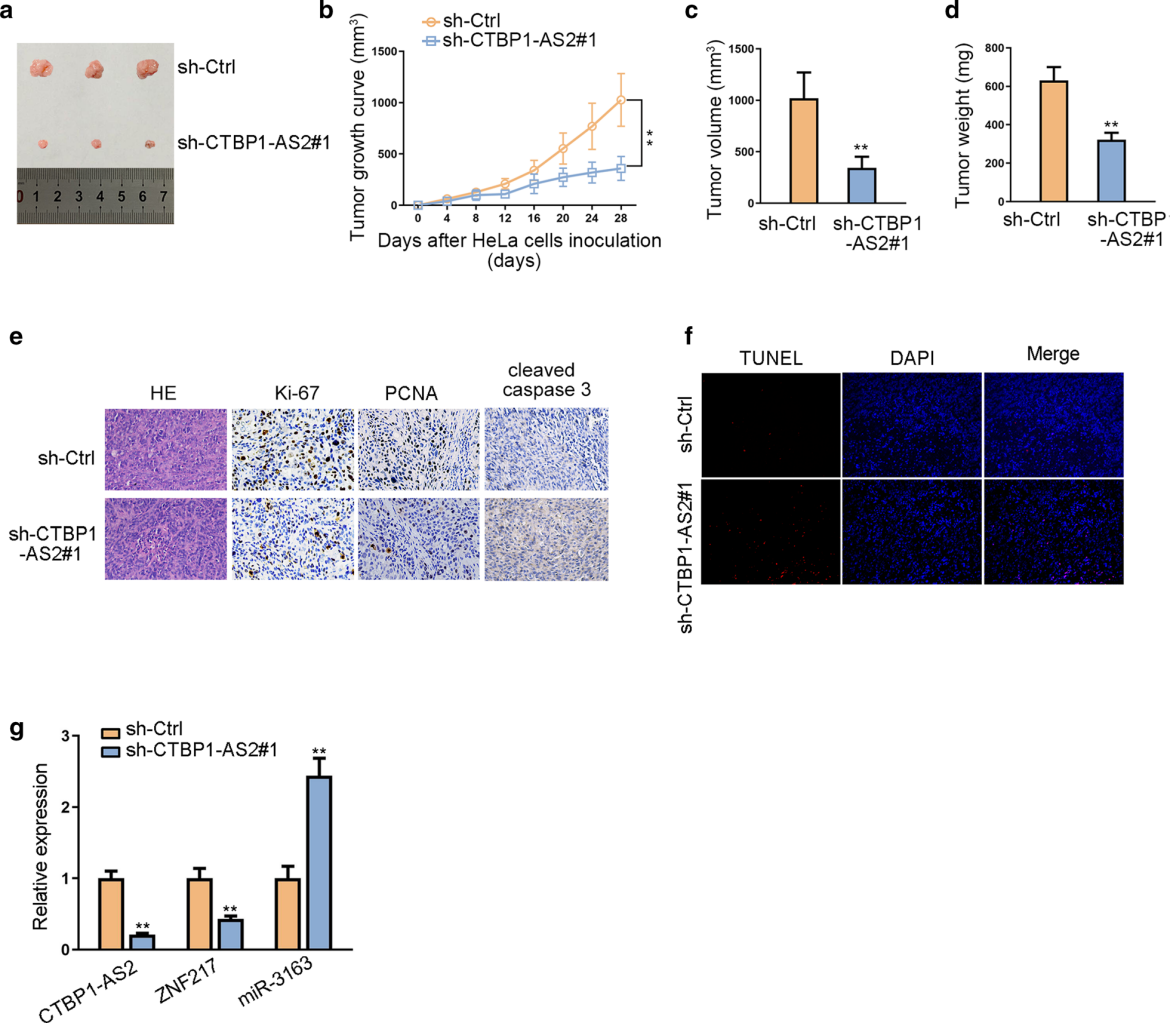
CTBP1-AS2/miR-3163/ZNF217 axis accelerates in vivo tumor growth. **a** Tumors derived from mice in two different groups were presented. **b**–**d** Volume and weight of tumors obtained from two groups were measured and shown. **e** HE staining and IHC staining of Ki-67 and cleaved caspase-3 in two groups. **f** TUNEL assay determined the positive effect of CTBP1-AS2 silencing on apoptosis. **g** qRT-PCR assay revealed the expression of CTBP1-AS2, miR-3163 and ZNF217 in tissues obtained from sh-CTBP1-AS2#1 group or sh-Ctrl group. ^**^P < 0.01

**Fig. 6:**
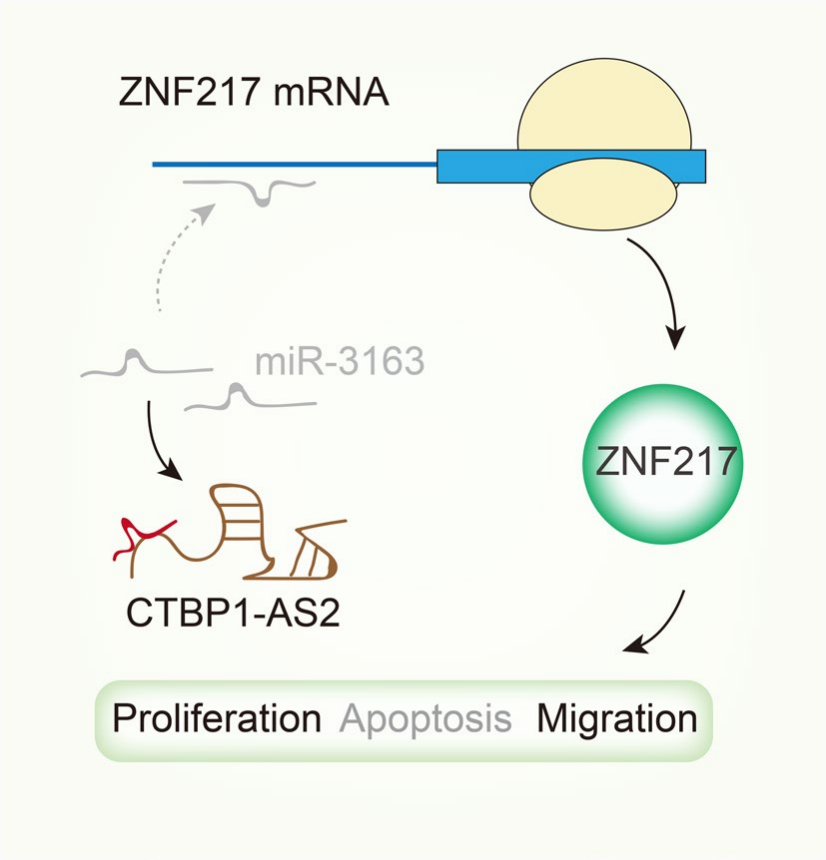
A schematic diagram illustrated that CTBP1-AS2 exerts its oncogenic properties in CC via up-regulating the expression of ZNF217 through sponging miR-3163

Xenografts model was used to further explore the tumor suppressing effects of sh-CTBP1-AS2 in vivo. We observed that the tumor growth speed was slower on nude mice after subcutaneously injection with sh-CTBP1-AS2#1 than control group (Fig. 5a). Besides, we found that the tumor volume and weight were apparently lessened in sh-CTBP1-AS2#1 group compared with control group (Fig. 5b–d). Moreover, as exhibited in Fig. 5e, positivity of Ki-67 was reduced and that of cleaved caspase-3 was enhanced by silenced CTBP1-AS2. TUNEL assay further determined the positive effect of CTBP1-AS2 silencing on apoptosis (Fig. 5f). Additionally, the levels of CTBP1-AS2 and ZNF217 were lower in tumor tissues removed from mice in sh-CTBP1-AS2#1 group than that in sh-Ctrl group (Fig. 5g). The level of miR-3163 was relatively higher in sh-CTBP1-AS2#2 group. These in vivo experiments demonstrated that knockdown of CTBP1-AS2 inhibited CC tumor growth in vivo. Taken together, CTBP1-AS2 exerted its oncogenic properties in CC via up-regulating the expression of ZNF217 through sponging miR-3163 (Fig. 6).

### Clinical relevance of CTBP1-AS2/miR-3163/ZNF217 axis with CC patients

In addition to in vitro and in vivo experiments, the clinical significance of CTBP1-AS2/miR-3163/ZNF217 axis was also analyzed. As presented in Additional file 4: Figure S3A, CTBP1-AS2 was expressed at a high level in CC tissues. Then, we divided CTBP1-AS2 into high or low expression of CTBP1-AS2 based on the medium expression value. The Kaplan–Meier survival analysis was conducted and the results depicted that high expression of CTBP1-AS2 led to unfavorable prognosis of CC patients (Additional file 4: Figure S3B). Besides, miR-3163 was significantly down-regulated in CC tissues (Additional file 4: Figure S3C) and up-regulation of miR-3163 predicted favorable prognosis of CC patients (Additional file 4: Figure S3D). Our present study also verified the overexpression of ZNF217 in CC tissues (Additional file 4: Figure S3E) and validated that up-regulated ZNF217 predicted poor prognosis of CC patients (Additional file 4: Figure S3F).

## Discussion

Accumulating evidence has revealed that lncRNAs play vital role in physiological activities and pathological variation [29]. Alteration of lncRNA expression is accompanied with the onset, development and progression of many cancers, including CC [30]. In the present study, CTBP1-AS2, a newly discovered lncRNA, was significantly up-regulated in CC tissues or cells compared with control tissues or cells. Knockdown of CTBP1-AS2 could inhibit CC cell proliferation and stimulated cell apoptosis. Furthermore, knockdown of CTBP1-AS2 could dampen CC cell migration and invasion ability to a large extent. These findings indicated that CTBP1-AS2 facilitates CC progression.

The competing endogenous RNAs (ceRNAs) network has been extensively reported, which revealed that lncRNAs could indirectly regulate the expression of mRNAs via binding to shared miRNAs [31]. This lncRNA-miRNA-mRNA network manifested a new way of RNA interaction and played an important role in tumor progression [32]. In this study, miR-3163 was identified to be sponged by CTBP1-AS2 through mechanism assays. We detected a significant down-regulation of miR-3163 in CC cell lines compared with non-tumor cervical epithelial cell line. Rescue experiments discovered that miR-3163 inhibition could abrogate the anti-tumor effects of CTBP1-AS2 knockdown on CC proliferation, apoptosis, migration and invasion. This represented that CTBP1-AS2 served as miR-3163 sponge in CC and its oncogenic function could be rescued by inhibiting miR-3163.

MiRNAs can bind to 3′UTR of downstream target gene with micro response elements (MREs) and suppress the function of target gene. We found that ZNF217 expression showed the most significant decrease after transfection with miR-3163 mimics. Then, we proved the physical interaction between miR-3163 and ZNF217. ZNF217 is essential for cell proliferation. It has been reported to be an oncogene in some cancers [33]. In this study, we observed that ZNF217 was also aberrantly up-regulated in CC cell lines, which is in line with the findings of some reports [34]. Functional experiments demonstrated that ZNF217 knockdown could inhibit cell proliferation, migration and invasion, yet promote apoptosis. Rescue experiments results manifested that ZNF217 overexpression could abolish the anti-tumor ability of sh-CTBP1-AS2. Collectively, these finding initially suggested that lncRNA CTBP1-AS2 deteriorated CC progression via up-regulating ZNF217 by acting as a miR-3163 sponge.

LncRNAs can exert functions in various biological processes through regulating their multiple downstream targets. PI3K/AKT and Src/FAK pathways are known as biological participant in cancer cell growth and metastasis [35–38]. In our current study, we determined that CTBP1-AS2 activated both PI3K/AKT signaling pathway through upregulating ZNF217.

The expression levels of CTBP1-AS2, miR-3163 and ZNF217 were different in CC cells. However, the specific mechanism leading to the differences remains unclear, which will be the elucidated in our future study. Lack of thorough investigation on the upstream molecular mechanism of CTBP1-AS2 is a limitation of our current study.

## Conclusion

In conclusion, CTBP1-AS2 expression was significantly overexpressed in CC. We identified a novel CTBP1-AS2/miR-3163/ZNF217 network in CC. The discovery of CTBP1-AS2 as an oncogene in CC progression could promisingly be used as a potential biomarker for CC patients.

## Supplementary information

**Additional file 1: Table S1.** Sequences of shRNAs, miRNA mimics/inhibitor and control miRNA, WT/mutant of CTBP1-AS2 and ZNF217, PCR primers.

**Additional file 2: Figure** S1. Upregulation of CTBP1-AS2 facilitates the malignant processes of H8 cell. A. CTBP1-AS2 was overexpressed in H8 cells by transfecting with CTBP1-AS2 expression vector. B-C. Cell proliferation was measured by CCK-8 and EdU assays. D. TUNEL assay was applied to analyze the apoptosis of H8 cells under the overexpression of CTBP1-AS2. E-F. The migration and invasion were detected in H8 cells after overexpression of CTBP1-AS2 by transwell assays. ^**^P < 0.01.

**Additional file 3: Figure S2.** CTBP1-AS2 activates PI3K/AKT and Src/FAK signaling pathways in CC cells by upregulating ZNF217. A. Cell cycle distribution was examined in two CC cells transfected with sh-Ctrl, sh-CTBP1-AS2#1, or co-transfected with sh-CTBP1-AS2#1 and pcDNA3.1/ZNF217. B. The levels of proliferation maker PCNA and cell cycle-related proteins were evaluated by western blot analysis. C-D. Western blot analysis of PI3K/AKT pathway related proteins (p-PI3K, PI3K, p-AKT, AKT, p-mTOR, mTOR, p-Src, Src, p-FAK, FAK) and Src/FAK pathway related proteins. ^*^P < 0.05.

**Additional file 4: Figure S3.** Clinical relevance of CTBP1-AS2/miR-3163/ZNF217 axis with CC patients. A. Expression of CTBP1-AS2 in CC tissues and normal tissues was revealed by qRT-PCR assay. B. Kaplan–Meier survival analysis of high expression or low expression of CTBP1-AS2 in CC patients. C. Expression of miR-3163 in CC tissues and normal tissues was revealed by qRT-PCR assay. D. Kaplan–Meier survival analysis of high expression or low expression of miR-3163 in CC patients. E. Expression of ZNF217 in CC tissues and normal tissues was revealed by qRT-PCR assay. F. Kaplan–Meier survival analysis of high expression or low expression of ZNF217 in CC patients. ^**^P < 0.01.

## Data Availability

Research data and material are not shared.

## Abbreviations

CC: Cervical cancer
ZNF217: Zinc finger protein 217
ncRNAs: Non-coding RNAs
lncRNAs: long non-coding RNAs
CCK-8: Cell counting kit-8
FISH: Fluorescence in situ hybridization Assay
WT: Wild type
MUT: Mutant
3′UTR: 3′ Un-translated region
RIP: RNA immunoprecipitation
RCSCs: Retinoblastoma Cancer Stem Cells
PCa: Prostate cancer
ceRNAs: Competing endogenous RNAs

## References

[1] Bray F (2018). Global cancer statistics 2018: GLOBOCAN estimates of incidence and mortality worldwide for 36 cancers in 185 countries. CA Cancer J Clin.

[2] Cohen PA (2019). Cervical cancer. Lancet.

[3] Siegel RL, Miller KD, Jemal A (2019). Cancer statistics, 2019. CA Cancer J Clin.

[4] Chen W (2017). Cancer incidence and mortality in China, 2013. Cancer Lett.

[5] Pan R (2017). Cancer incidence and mortality: a cohort study in China, 2008–2013. Int J Cancer.

[6] Pimple SA, Mishra GA (2019). Global strategies for cervical cancer prevention and screening. Minerva Ginecol.

[7] Singh SV (2015). Proteasomal inhibition sensitizes cervical cancer cells to mitomycin C-induced bystander effect: the role of tumor microenvironment. Cell Death Dis.

[8] Regalado Porras GO, Chavez Nogueda J, Poitevin Chacon A (2018). Chemotherapy and molecular therapy in cervical cancer. Rep Pract Oncol Radiother.

[9] Li H, Wu X, Cheng X (2016). Advances in diagnosis and treatment of metastatic cervical cancer. J Gynecol Oncol.

[10] Derks M (2018). Surgical treatment of early-stage cervical cancer: a multi-institution experience in 2124 cases in The Netherlands over a 30-year period. Int J Gynecol Cancer.

[11] Lu S (2019). A hidden human proteome encoded by ‘non-coding’ genes. Nucleic Acids Res.

[12] Wang J, Cai H, Dai Z, Wang G (2019). Correction: downregulation of LncRNA XIST inhibits cell proliferation via regulating miR-744/RING1 axis in non-small cell lung cancer. Clin Sci.

[13] Dai JH (2019). Silencing of long noncoding RNA SBF2-AS1 inhibits proliferation, migration and invasion and contributes to apoptosis in osteosarcoma cells by upregulating microRNA-30a to suppress FOXA1 expression. Cell Cycle.

[14] Wang N (2019). Long noncoding RNA DANCR regulates proliferation and migration by epigenetically silencing FBP1 in tumorigenesis of cholangiocarcinoma. Cell Death Dis.

[15] Sassenberg M (2019). Upregulation of the long non-coding RNA CASC9 as a biomarker for squamous cell carcinoma. BMC Cancer.

[16] Jiang L, Li Z, Wang R (2019). Long noncoding RNAs in lung cancer: regulation patterns, biologic function and diagnosis implications (Review). Int J Oncol.

[17] Mao BD (2019). LINC00511 knockdown prevents cervical cancer cell proliferation and reduces resistance to paclitaxel. J Biosci.

[18] Ma B (2016). Long intergenic non-coding RNA 271 is predictive of a poorer prognosis of papillary thyroid cancer. Sci Rep.

[19] Muhammad N (2016). Anti-miR-203 suppresses ER-positive breast cancer growth and stemness by targeting SOCS3. Oncotarget.

[20] Liu DL (2020). miR-17-5p and miR-20a-5p suppress postoperative metastasis of hepatocellular carcinoma via blocking HGF/ERBB3-NF-κB positive feedback loop. Theranostics.

[21] Lu Z (2019). microRNA-17 functions as an oncogene by downregulating Smad3 expression in hepatocellular carcinoma. Cell Death Dis.

[22] Yang B (2019). MicroRNA-3163 targets ADAM-17 and enhances the sensitivity of hepatocellular carcinoma cells to molecular targeted agents. Cell Death Dis.

[23] Ren H (2020). Long noncoding MAGI2-AS3 promotes colorectal cancer progression through regulating miR-3163/TMEM106B axis. J Cell Physiol.

[24] Si W (2019). The coordination between ZNF217 and LSD1 contributes to hepatocellular carcinoma progress and is negatively regulated by miR-101. Exp Cell Res.

[25] Ma XR (2019). Long non-coding RNA SNHG15 accelerates the progression of non-small cell lung cancer by absorbing miR-211-3p. Eur Rev Med Pharmacol Sci.

[26] Jia M (2016). Silencing of ABCG2 by MicroRNA-3163 Inhibits Multidrug Resistance in Retinoblastoma Cancer Stem Cells. J Korean Med Sci.

[27] Jiang X (2016). Elevated expression of ZNF217 promotes prostate cancer growth by restraining ferroportin-conducted iron egress. Oncotarget.

[28] Bellanger A (2017). The critical role of the ZNF217 oncogene in promoting breast cancer metastasis to the bone. J Pathol.

[29] Fatica A, Bozzoni I (2014). Long non-coding RNAs: new players in cell differentiation and development. Nat Rev Genet.

[30] Sharma S, Munger K (2018). Expression of the cervical carcinoma expressed PCNA regulatory (CCEPR) long noncoding RNA is driven by the human papillomavirus E6 protein and modulates cell proliferation independent of PCNA. Virology.

[31] Salmena L (2011). A ceRNA hypothesis: the Rosetta Stone of a hidden RNA language?. Cell.

[32] Zhang Y (2016). Comprehensive characterization of lncRNA-mRNA related ceRNA network across 12 major cancers. Oncotarget.

[33] Li Z (2015). MiR-203 suppresses ZNF217 upregulation in colorectal cancer and its oncogenicity. PLoS ONE.

[34] Littlepage LE (2012). The transcription factor ZNF217 is a prognostic biomarker and therapeutic target during breast cancer progression. Cancer Discov.

[35] Liang Y (2018). CX3CL1 involves in breast cancer metastasizing to the spine via the Src/FAK signaling pathway. J Cancer.

[36] Wen J (2019). STAT3-induced upregulation of lncRNA ABHD11-AS1 promotes tumour progression in papillary thyroid carcinoma by regulating miR-1301-3p/STAT3 axis and PI3K/AKT signalling pathway. Cell Prolif.

[37] Huang Y (2017). LncRNA AK023391 promotes tumorigenesis and invasion of gastric cancer through activation of the PI3K/Akt signaling pathway. J. Exp Clin Cancer Res..

[38] Liu H (2018). Invasion-related circular RNA circFNDC3B inhibits bladder cancer progression through the miR-1178-3p/G3BP2/SRC/FAK axis. Mol Cancer.

