# Supplementary material for: Long non-coding RNA CTBP1-AS2 enhances cervical cancer progression via up-regulation of ZNF217 through sponging miR-3163
Source: Cancer Cell Int. 2020 Jul 28;20:343. doi: 10.1186/s12935-020-01430-5 (PMC7388209; doi:10.1186/s12935-020-01430-5)
Supplement: Supplementary file 1 — Additional file 1: Table S1. Sequences of shRNAs, miRNA mimics/inhibitor and control miRNA, WT/mutant of CTBP1-AS2 and ZNF217, PCR primers. [file 12935_2020_1430_MOESM1_ESM.docx]

**Sequences of shRNAs of CTBP1-AS2, shRNAs of ZNF217, miRNA mimics/inhibitor and control miRNA**

sh-CTBP1-AS2#1

5’-CCGGCTGAGTGTCCAGCAAACATCACTCGAGTGATGTTTGCT

GGACACTCAGTTTTTTG-3’

sh-CTBP1-AS2#2

5’-CCGGGCTCAGTCCAGAAGAACAGTTCTCGAGAACTGTTCTT

CTGGACTGAGCTTTTTTG-3’

sh-ZNF217#1

5’-CCGGACACATTCAGAAGACCTTAATCTCGAGATTAAGGTCTT

CTGAATGTGTTTTTTG-3’

sh-ZNF217#2

5’-CCGGTAGAACCAAGGCGGATGTTACCTCGAGGTAACATCCG

CCTTGGTTCTATTTTTG-3’

MiR-3163 mimics

5’-UAUAAAAUGAGGGCAGUAAGAC-3’

MiR-3163 inhibitor

5’-GUCUUACUGCCCUCAUUUUAUA-3’

Control miRNA

5’-GACACAUAAUGAAUAGUGAGGA-3’

**Sequences of WT and MUT of CTBP1-AS2 and ZNF217 used in luciferase reporter assay**

WT of CTBP1-AS2

5’-CCGCCCACGTCAGCGCCTGGCGCGGGCCCGAACAAACCACG

GCATGGAGACGGAATGATGCAGACTCCTATCCGCGAGATACCTGCTGTAATTTCGCTGTGGTTCGTGAGGACGCGTCCTGGGCTGCCCTGAGAAGCCTACGTCTCCCCTTCGAGGCCCGGGAAGACCTCCAACCCCGCTGACAATGCTGGGCCCTCAGCCAGACCTGCCCTGCGTGCCACGTTCTGTTCTAAGATCGGGCTGCCGAGCTGTGGCCTGGAGGAAACCTGGCAGGTGTCACCAGAGCTTTGGGGAGGGTGAGGCCCTGTCGGGGAAGCCCAGTGGGAGTCATGAAGGAGGGAACACGTGTGGAGCCCTTGTAGGGGGAGGGGCAGCCCTGCAGAGATCTAAGAAAAAATTCCAGAAAAATGAGCAGCAACCTCCAAGGCCAGGCATCGGTGCAGGGGACAAGGGGTCGTAGCTGGAGGGGCCTAGGTGAGGCTGCCGGGAAGGGACGATGTGGTTGGTGGAGTGCACAGGCAGGGACCTCACTGGACTTTCCTGTCTGCTCAGCATGGACAGGCAGCCCAGGAGAAGGCAGCACGTGGCCGGGTGCAGGGACGTACCACCCCCACTTCCCCAGGGGAGCTGGGGTCAGACGAGTCCCAGGCACTCCATCCTCTGCAGCAAGTCAGGTTGTGATTTACTAGGGGGTGGTGAATATAATGGAGAGACTTCTGGGGAGGAATTCCTGGCTCCCGCGTGGACTTGCAGAGCTCAACAGGCAGCCACGTGGCTGAGTGTCCAGCAAACATCACATAAGGCTTTGGGTCCTGCAGGTGGGGCTGCCATGAGCAGCAAGCTCAGTCCAGAAGAACAGTTCCTCTCCAGGATCCACTTCCTGCGCACTTTTATGTGCAGTGTAGCTGGAGCAGAGCTCCCCGGAATTCCACAGGCAACTGAGAACGGAGAGGGATGCAGGCCAGCCAGGGATCCAGCGTCTTCCCCATCGTCACTCTCCATGGCCTCCGTCTACACACAGTGTTCGTCTGCACAGCTTGTCAGCGCGTTATCATGACTTCTATTTGGCACCGGCCCGTCTGTCCACTGTCCTGGCTTGTTCCAAGCGCTGCCTTCTCCAACTGGGGTCCTGGCTGCAGAGCTGTCTGCTCCCCACGTTGGGCAACTCCAGCCAAGATTCCTACACCCAAATGTGACCGTGTTGCTCACGAAGAAGGCTCAGCTTTGCGTGTGCCCAGCCGTGTGCACAGCTCGTCCCAACTCCTGCGGGTGGCACCTGCCTCTCCCACCTCCAGTCTTTCCCCTGTGATGAGCAGATGACCACCGCCCTCCAGGGTCAGCGTTTGGCTGTTTGTGTGCCTGCCCACCCGCCTCCCTGTGCCTGGGCCTGCCCTGTGCCTGCACACAGGAGTGGCTCATGGCGTTTGCCCTACACGGATGGGCTGTCCTGGGAGCTACTGGACAGTCACCTTGGTGGGAATGCCAGAGGCATGGGCATTAGGTCCCCCCGGCCAGCCTCCGTTGCCACATGGGCTATTTTTGTCCATCGCGTGGGACAACCTAGTATTGGGGGAAAACTCAGTCCACTCTAAAGAAGCATCGGCGTTTTGGATGGACGAATGCTGCTTCATGCGACTCCATGTCAATGGACTATTTTATTCAACTCGGGTATTTATGAGTGTCTCCTCTGTGCCAGCCACGTGTGAAAGAAGAACTCCAAGTTACTGGACACCAGGACAAACCAGGTAGATCACTGCATCCGGTTCCTCCTCTTTCTGCCGACTCTCCCGGGCAACCGTTCTGATTCCTGGCATGGAGTTGGGGGCGAGCTCATCAGTACCCAGAGCGCCTGGTGTGCAGCCAAAGGCCCCCATTTTATTAGTCGGTGTGTTCAGTATCACGCTCAAGACAAGGGCCAACCTATTACTTCACACACCTCAAATACAAAAAGTGCCTGGGAACGTCGATGAGGTATTAAAGGAAACCACTGGTCTTGTCAGTCATGTACACACAGAAAAAAAAACCAGTCATTTTTATTTTTATTTTTTGAGACAGAGTTTCGCTCTTGTTGCCCAGGCTGGAGTGCAATGGTGCAATTTCAGCTCACTGCAATCTCTGCCACCCGGATTCAAGCAATTCTCCTGTCTCAGCCTCCCGAGTAGCTGGAATTGCAGACACCCATCACCACGCCCAGCTAATTTTATGTATTTTTAGTAGAGATGGGGTTTCACCATGTTGGTCAGGCTGGTCTTGAACTCCTGACCTCAGGTGATCCACCCACGTCAGGCTCCCAAAGTGCTGAGATTACAGGCATGAACCACTGTGCCCAGCCGAAAACAACCAAGAATTTTTTAACAATAAAATGACCAGCAAGTAAACTTGAAACAAAAATGGACAAATATCTAAAATATTTTTAAATGTATGTATTTTAATTGGCAAATAATACTTGTATATATTGTTTACAACTTGTTTTAAAGTATGAATACATTGTGGAATGGCTAAATCAAGTTAATTAACATGCATTAACTCACATACTTTTTGTTTTTTGGTGTGGTGAGGACACTTAACATCTACTCTCTTAGCAATTTTCCAGTCACCATGTCCTACTATAGATCCCTAGAACATATTCCTCCAATGTAACTGAAATGTATCCCTTGACCAACATCACTGCCCGGCCCCAGCCCCAGGTAAACACCATTCTACCCTCTGCTTCTGTGAGTTCAGCTTTTCTAGATTTCACCTGTGAGTGAGATTGTGCAGCATTTGTCTTTCTGTGCCTGGGTTTTTTCACATAATGCCCTTCTGGTTCATCTATATTGTCATATATGGTAGCATTGTCTTCCTTTGTAAGGCTGACTGGTATACCATTGCGTATATACACCACATTGTTTACCTAAAATATTTTTTAATACAATCGCTGAAAACTGCCAAAAAAAAAAAAAAAA-3’

MUT of CTBP1-AS2

5’-CCGCCCACGTCAGCGCCTGGCGCGGGCCCGAACAAACCACG

GCATGGAGACGGAATGATGCAGACTCCTATCCGCGAGATACCTGCTGTAATTTCGCTGTGGTTCGTGAGGACGCGTCCTGGGCTGCCCTGAGAAGCCTACGTCTCCCCTTCGAGGCCCGGGAAGACCTCCAACCCCGCTGACAATGCTGGGCCCTCAGCCAGACCTGCCCTGCGTGCCACGTTCTGTTCTAAGATCGGGCTGCCGAGCTGTGGCCTGGAGGAAACCTGGCAGGTGTCACCAGAGCTTTGGGGAGGGTGAGGCCCTGTCGGGGAAGCCCAGTGGGAGTCATGAAGGAGGGAACACGTGTGGAGCCCTTGTAGGGGGAGGGGCAGCCCTGCAGAGATCTAAGAAAAAATTCCAGAAAAATGAGCAGCAACCTCCAAGGCCAGGCATCGGTGCAGGGGACAAGGGGTCGTAGCTGGAGGGGCCTAGGTGAGGCTGCCGGGAAGGGACGATGTGGTTGGTGGAGTGCACAGGCAGGGACCTCACTGGACTTTCCTGTCTGCTCAGCATGGACAGGCAGCCCAGGAGAAGGCAGCACGTGGCCGGGTGCAGGGACGTACCACCCCCACTTCCCCAGGGGAGCTGGGGTCAGACGAGTCCCAGGCACTCCATCCTCTGCAGCAAGTCAGGTTGTGATTTACTAGGGGGTGGTGAATATAATGGAGAGACTTCTGGGGAGGAATTCCTGGCTCCCGCGTGGACTTGCAGAGCTCAACAGGCAGCCACGTGGCTGAGTGTCCAGCAAACATCACATAAGGCTTTGGGTCCTGCAGGTGGGGCTGCCATGAGCAGCAAGCTCAGTCCAGAAGAACAGTTCCTCTCCAGGATCCACTTCCTGCGCACTTTTATGTGCAGTGTAGCTGGAGCAGAGCTCCCCGGAATTCCACAGGCAACTGAGAACGGAGAGGGATGCAGGCCAGCCAGGGATCCAGCGTCTTCCCCATCGTCACTCTCCATGGCCTCCGTCTACACACAGTGTTCGTCTGCACAGCTTGTCAGCGCGTTATCATGACTTCTATTTGGCACCGGCCCGTCTGTCCACTGTCCTGGCTTGTTCCAAGCGCTGCCTTCTCCAACTGGGGTCCTGGCTGCAGAGCTGTCTGCTCCCCACGTTGGGCAACTCCAGCCAAGATTCCTACACCCAAATGTGACCGTGTTGCTCACGAAGAAGGCTCAGCTTTGCGTGTGCCCAGCCGTGTGCACAGCTCGTCCCAACTCCTGCGGGTGGCACCTGCCTCTCCCACCTCCAGTCTTTCCCCTGTGATGAGCAGATGACCACCGCCCTCCAGGGTCAGCGTTTGGCTGTTTGTGTGCCTGCCCACCCGCCTCCCTGTGCCTGGGCCTGCCCTGTGCCTGCACACAGGAGTGGCTCATGGCGTTTGCCCTACACGGATGGGCTGTCCTGGGAGCTACTGGACAGTCACCTTGGTGGGAATGCCAGAGGCATGGGCATTAGGTCCCCCCGGCCAGCCTCCGTTGCCACATGGGCTATTTTTGTCCATCGCGTGGGACAACCTAGTATTGGGGGAAAACTCAGTCCACTCTAAAGAAGCATCGGCGTTTTGGATGGACGAATGCTGCTTCATGCGACTCCATGTCAATGGACTTAAAATATCAACTCGGGTATTTATGAGTGTCTCCTCTGTGCCAGCCACGTGTGAAAGAAGAACTCCAAGTTACTGGACACCAGGACAAACCAGGTAGATCACTGCATCCGGTTCCTCCTCTTTCTGCCGACTCTCCCGGGCAACCGTTCTGATTCCTGGCATGGAGTTGGGGGCGAGCTCATCAGTACCCAGAGCGCCTGGTGTGCAGCCAAAGGCCCCCTAAAATATAGTCGGTGTGTTCAGTATCACGCTCAAGACAAGGGCCAACCTATTACTTCACACACCTCAAATACAAAAAGTGCCTGGGAACGTCGATGAGGTATTAAAGGAAACCACTGGTCTTGTCAGTCATGTACACACAGAAAAAAAAACCAGTCATTTTTATTTTTATTTTTTGAGACAGAGTTTCGCTCTTGTTGCCCAGGCTGGAGTGCAATGGTGCAATTTCAGCTCACTGCAATCTCTGCCACCCGGATTCAAGCAATTCTCCTGTCTCAGCCTCCCGAGTAGCTGGAATTGCAGACACCCATCACCACGCCCAGCTATAAAATAGTATTTTTAGTAGAGATGGGGTTTCACCATGTTGGTCAGGCTGGTCTTGAACTCCTGACCTCAGGTGATCCACCCACGTCAGGCTCCCAAAGTGCTGAGATTACAGGCATGAACCACTGTGCCCAGCCGAAAACAACCAAGAATTTTTTAACAATAAAATGACCAGCAAGTAAACTTGAAACAAAAATGGACAAATATCTAAAATATTTTTAAATGTATGTATTTTAATTGGCAAATAATACTTGTATATATTGTTTACAACTTGTTTTAAAGTATGAATACATTGTGGAATGGCTAAATCAAGTTAATTAACATGCATTAACTCACATACTTTTTGTTTTTTGGTGTGGTGAGGACACTTAACATCTACTCTCTTAGCAATTTTCCAGTCACCATGTCCTACTATAGATCCCTAGAACATATTCCTCCAATGTAACTGAAATGTATCCCTTGACCAACATCACTGCCCGGCCCCAGCCCCAGGTAAACACCATTCTACCCTCTGCTTCTGTGAGTTCAGCTTTTCTAGATTTCACCTGTGAGTGAGATTGTGCAGCATTTGTCTTTCTGTGCCTGGGTTTTTTCACATAATGCCCTTCTGGTTCATCTATATTGTCATATATGGTAGCATTGTCTTCCTTTGTAAGGCTGACTGGTATACCATTGCGTATATACACCACATTGTTTACCTAAAATATTTTTTAATACAATCGCTGAAAACTGCCAAAAAAAAAAAAAAAA-3’

WT of ZNF217

5’-ATGCAATCGAAAGTGACAGGAAACATGCCAACTCAATCCCTC

TTAATGTACATGGATGGGCCAGAAGTGATTGGCAGCTCTCTTGGCAGTCCGATGGAGATGGAGGATGCCTTGTCAATGAAAGGGACCGCTGTTGTTCCATTCCGAGCTACACAAGAAAAAAATGTCATCCAAATCGAGGGGTATATGCCCTTGGATTGCATGTTCTGCAGCCAGACCTTCACACATTCAGAAGACCTTAATAAACATGTCTTAATGCAACACCGGCCTACCCTCTGTGAACCAGCAGTTCTTCGGGTTGAAGCAGAGTATCTCAGTCCGCTTGATAAAAGTCAAGTGCGAACAGAACCTCCCAAGGAAAAGAATTGCAAGGAAAATGAATTTAGCTGTGAGGTATGTGGGCAGACATTTAGAGTCGCTTTTGATGTTGAGATCCACATGAGAACACACAAAGATTCTTTCACTTACGGGTGTAACATGTGCGGAAGAAGATTCAAGGAGCCTTGGTTTCTTAAAAATCACATGCGGACACATAATGGCAAATCGGGGGCCAGAAGCAAACTGCAGCAAGGCTTGGAGAGTAGTCCAGCAACGATCAACGAGGTCGTCCAGGTGCACGCGGCCGAGAGCATCTCCTCTCCTTACAAAATCTGCATGGTTTGTGGCTTCCTATTTCCAAATAAAGAAAGTCTAATTGAGCACCGCAAGGTGCACACCAAAAAAACTGCTTTCGGTACCAGCAGCGCGCAGACAGACTCTCCACAAGGAGGAATGCCGTCCTCGAGGGAGGACTTCCTGCAGTTGTTCAACTTGAGACCAAAATCTCACCCTGAAACGGGGAAGAAGCCTGTCAGATGCATCCCTCAGCTCGATCCGTTCACCACCTTCCAGGCTTGGCAGCTGGCTACCAAAGGAAAAGTTGCCATTTGCCAAGAAGTGAAGGAATCGGGGCAAGAAGGGAGCACCGACAACGACGATTCGAGTTCCGAGAAGGAGCTTGGAGAAACAAATAAGGGCAGTTGTGCAGGCCTCTCGCAAGAGAAAGAGAAGTGCAAACACTCCCACGGCGAAGCGCCCTCCGTGGACGCGGATCCCAAGTTACCCAGTAGCAAGGAGAAGCCCACTCACTGCTCCGAGTGCGGCAAAGCTTTCAGAACCTACCACCAGCTGGTCTTGCACTCCAGGGTCCACAAGAAGGACCGGAGGGCCGGCGCGGAGTCGCCCACCATGTCTGTGGACGGGAGGCAGCCGGGGACGTGTTCTCCTGACCTCGCCGCCCCTCTGGATGAAAATGGAGCCGTGGATCGAGGGGAAGGTGGTTCTGAAGACGGATCTGAGGATGGGCTTCCCGAAGGAATCCATCTGGATAAAAATGATGATGGAGGAAAAATAAAACATCTTACATCTTCAAGAGAGTGTAGTTATTGTGGAAAGTTTTTCCGTTCAAATTATTACCTCAATATTCATCTCAGAACGCATACAGGTGAAAAACCATACAAATGTGAATTTTGTGAATATGCTGCAGCCCAGAAGACATCTCTGAGGTATCACTTGGAGAGACATCACAAGGAAAAACAAACCGATGTTGCTGCTGAAGTCAAGAACGATGGTAAAAATCAGGACACTGAAGATGCACTATTAACCGCTGACAGTGCGCAAACCAAAAATTTGAAAAGATTTTTTGATGGTGCCAAAGATGTTACAGGCAGTCCACCTGCAAAGCAGCTTAAGGAGATGCCTTCTGTTTTTCAGAATGTTCTGGGCAGCGCTGTCCTCTCACCAGCACACAAAGATACTCAGGATTTCCATAAAAATGCAGCTGATGACAGTGCTGATAAAGTGAATAAAAACCCTACCCCTGCTTACCTGGACCTGTTAAAAAAGAGATCAGCAGTTGAAACTCAGGCAAATAACCTCATCTGTAGAACCAAGGCGGATGTTACTCCTCCTCCGGATGGCAGTACCACCCATAACCTTGAAGTTAGCCCCAAAGAGAAGCAAACGGAGACCGCAGCTGACTGCAGATACAGGCCAAGTGTGGATTGTCACGAAAAACCTTTAAATTTATCCGTGGGGGCTCTTCACAATTGCCCGGCAATTTCTTTGAGTAAAAGTTTGATTCCAAGTATCACCTGTCCATTTTGTACCTTCAAGACATTTTATCCAGAAGTTTTAATGATGCACCAGAGACTGGAGCATAAATACAATCCTGACGTTCATAAAAACTGTCGAAACAAGTCCTTGCTTAGAAGTCGACGTACCGGATGCCCGCCAGCGTTGCTGGGAAAAGATGTGCCTCCCCTCTCTAGTTTCTGTAAACCCAAGCCCAAGTCTGCTTTCCCGGCGCAGTCCAAATCCCTGCCATCTGCGAAGGGGAAGCAGAGCCCTCCTGGGCCAGGCAAGGCCCCTCTGACTTCAGGGATAGACTCTAGCACTTTAGCCCCAAGTAACCTGAAGTCCCACAGACCACAGCAGAATGTGGGGGTCCAAGGGGCCGCCACCAGGCAACAGCAATCTGAGATGTTTCCTAAAACCAGTGTTTCCCCTGCACCGGATAAGACAAAAAGACCCGAGACAAAATTGAAACCTCTTCCAGTAGCTCCTTCTCAGCCCACCCTCGGCAGCAGTAACATCAATGGTTCCATCGACTACCCCGCCAAGAACGACAGCCCGTGGGCACCTCCGGGAAGAGACTATTTCTGTAATCGGAGTGCCAGCAATACTGCAGCAGAATTTGGTGAGCCCCTTCCAAAAAGACTGAAGTCCAGCGTGGTTGCCCTTGACGTTGACCAGCCCGGGGCCAATTACAGAAGAGGCTATGACCTTCCCAAGTACCATATGGTCAGAGGCATCACATCACTGTTACCGCAGGACTGTGTGTATCCGTCGCAGGCGCTGCCTCCCAAACCAAGGTTCCTGAGCTCCAGCGAGGTCGATTCTCCAAATGTGCTGACTGTTCAGAAGCCCTATGGTGGCTCCGGGCCACTTTACACTTGTGTGCCTGCTGGTAGTCCAGCATCCAGCTCGACGTTAGAAGGAAAAAGGCCTGTGTCATATCAACACTTATCTAACAGCATGGCACAAAAGAGAAACTATGAGAATTTTATTGGGAATGCACATTATCGACCAAATGACAAAAAAACTTGA-3’

MUT of ZNF217

5’-ATGCAATCGAAAGTGACAGGAAACATGCCAACTCAATCCCTC

TTAATGTACATGGATGGGCCAGAAGTGATTGGCAGCTCTCTTGGCAGTCCGATGGAGATGGAGGATGCCTTGTCAATGAAAGGGACCGCTGTTGTTCCATTCCGAGCTACACAAGAAAAAAATGTCATCCAAATCGAGGGGTATATGCCCTTGGATTGCATGTTCTGCAGCCAGACCTTCACACATTCAGAAGACCTTAATAAACATGTCTTAATGCAACACCGGCCTACCCTCTGTGAACCAGCAGTTCTTCGGGTTGAAGCAGAGTATCTCAGTCCGCTTGATAAAAGTCAAGTGCGAACAGAACCTCCCAAGGAAAAGAATTGCAAGGAAAATGAATTTAGCTGTGAGGTATGTGGGCAGACATTTAGAGTCGCTTTTGATGTTGAGATCCACATGAGAACACACAAAGATTCTTTCACTTACGGGTGTAACATGTGCGGAAGAAGATTCAAGGAGCCTTGGTTTCTTAAAAATCACATGCGGACACATAATGGCAAATCGGGGGCCAGAAGCAAACTGCAGCAAGGCTTGGAGAGTAGTCCAGCAACGATCAACGAGGTCGTCCAGGTGCACGCGGCCGAGAGCATCTCCTCTCCTTACAAAATCTGCATGGTTTGTGGCTTCCTATTTCCAAATAAAGAAAGTCTAATTGAGCACCGCAAGGTGCACACCAAAAAAACTGCTTTCGGTACCAGCAGCGCGCAGACAGACTCTCCACAAGGAGGAATGCCGTCCTCGAGGGAGGACTTCCTGCAGTTGTTCAACTTGAGACCAAAATCTCACCCTGAAACGGGGAAGAAGCCTGTCAGATGCATCCCTCAGCTCGATCCGTTCACCACCTTCCAGGCTTGGCAGCTGGCTACCAAAGGAAAAGTTGCCATTTGCCAAGAAGTGAAGGAATCGGGGCAAGAAGGGAGCACCGACAACGACGATTCGAGTTCCGAGAAGGAGCTTGGAGAAACAAATAAGGGCAGTTGTGCAGGCCTCTCGCAAGAGAAAGAGAAGTGCAAACACTCCCACGGCGAAGCGCCCTCCGTGGACGCGGATCCCAAGTTACCCAGTAGCAAGGAGAAGCCCACTCACTGCTCCGAGTGCGGCAAAGCTTTCAGAACCTACCACCAGCTGGTCTTGCACTCCAGGGTCCACAAGAAGGACCGGAGGGCCGGCGCGGAGTCGCCCACCATGTCTGTGGACGGGAGGCAGCCGGGGACGTGTTCTCCTGACCTCGCCGCCCCTCTGGATGAAAATGGAGCCGTGGATCGAGGGGAAGGTGGTTCTGAAGACGGATCTGAGGATGGGCTTCCCGAAGGAATCCATCTGGATAAAAATGATGATGGAGGAAAAATAAAACATCTTACATCTTCAAGAGAGTGTAGTTATTGTGGAAAGTTTTTCCGTTCAAATTATTACCTCAATATTCATCTCAGAACGCATACAGGTGAAAAACCATACAAATGTGAATTTTGTGAATATGCTGCAGCCCAGAAGACATCTCTGAGGTATCACTTGGAGAGACATCACAAGGAAAAACAAACCGATGTTGCTGCTGAAGTCAAGAACGATGGTAAAAATCAGGACACTGAAGATGCACTATTAACCGCTGACAGTGCGCAAACCAAAAATTTGAAAAGATTTTTTGATGGTGCCAAAGATGTTACAGGCAGTCCACCTGCAAAGCAGCTTAAGGAGATGCCTTCTGTTTTTCAGAATGTTCTGGGCAGCGCTGTCCTCTCACCAGCACACAAAGATACTCAGGATTTCCATAAAAATGCAGCTGATGACAGTGCTGATAAAGTGAATAAAAACCCTACCCCTGCTTACCTGGACCTGTTAAAAAAGAGATCAGCAGTTGAAACTCAGGCAAATAACCTCATCTGTAGAACCAAGGCGGATGTTACTCCTCCTCCGGATGGCAGTACCACCCATAACCTTGAAGTTAGCCCCAAAGAGAAGCAAACGGAGACCGCAGCTGACTGCAGATACAGGCCAAGTGTGGATTGTCACGAAAAACCTTTAAATTTATCCGTGGGGGCTCTTCACAATTGCCCGGCAATTTCTTTGAGTAAAAGTTTGATTCCAAGTATCACCTGTCCATTTTGTACCTTCAAGACAAAATACCAGAAGTTTTAATGATGCACCAGAGACTGGAGCATAAATACAATCCTGACGTTCATAAAAACTGTCGAAACAAGTCCTTGCTTAGAAGTCGACGTACCGGATGCCCGCCAGCGTTGCTGGGAAAAGATGTGCCTCCCCTCTCTAGTTTCTGTAAACCCAAGCCCAAGTCTGCTTTCCCGGCGCAGTCCAAATCCCTGCCATCTGCGAAGGGGAAGCAGAGCCCTCCTGGGCCAGGCAAGGCCCCTCTGACTTCAGGGATAGACTCTAGCACTTTAGCCCCAAGTAACCTGAAGTCCCACAGACCACAGCAGAATGTGGGGGTCCAAGGGGCCGCCACCAGGCAACAGCAATCTGAGATGTTTCCTAAAACCAGTGTTTCCCCTGCACCGGATAAGACAAAAAGACCCGAGACAAAATTGAAACCTCTTCCAGTAGCTCCTTCTCAGCCCACCCTCGGCAGCAGTAACATCAATGGTTCCATCGACTACCCCGCCAAGAACGACAGCCCGTGGGCACCTCCGGGAAGAGACTATTTCTGTAATCGGAGTGCCAGCAATACTGCAGCAGAATTTGGTGAGCCCCTTCCAAAAAGACTGAAGTCCAGCGTGGTTGCCCTTGACGTTGACCAGCCCGGGGCCAATTACAGAAGAGGCTATGACCTTCCCAAGTACCATATGGTCAGAGGCATCACATCACTGTTACCGCAGGACTGTGTGTATCCGTCGCAGGCGCTGCCTCCCAAACCAAGGTTCCTGAGCTCCAGCGAGGTCGATTCTCCAAATGTGCTGACTGTTCAGAAGCCCTATGGTGGCTCCGGGCCACTTTACACTTGTGTGCCTGCTGGTAGTCCAGCATCCAGCTCGACGTTAGAAGGAAAAAGGCCTGTGTCATATCAACACTTATCTAACAGCATGGCACAAAAGAGAAACTATGAGAATTTTATTGGGAATGCACATTATCGACCAAATGACAAAAAAACTTGA-3’

**PCR primer sequences：**

MiR-3163

Reverse transcription stem ring primer： CTCAACTGGTGTCGTGGAGTCGGCAATTCAGTTGAGCGTCTTAC

Forward primer

GCCGAGTATAAAATGAGGGCA

Reverse primer

CTCAACTGGTGTCGTGGA

MiR-3150b-3p

Reverse transcription stem ring primer： CTCAACTGGTGTCGTGGAGTCGGCAATTCAGTTGAGCCCAACCT

Forward primer

GCCGAGTGAGGAGATCGTCG

Reverse primer

CTCAACTGGTGTCGTGGA

MiR-4784

Reverse transcription stem ring primer： CTCAACTGGTGTCGTGGAGTCGGCAATTCAGTTGAGCTCAGTC

Forward primer

GCCGAGTGAGGAGATGCTGG

Reverse primer

CTCAACTGGTGTCGTGGA

U6

Forward primer

CTCGCTTCGGCAGCACA

Reverse primer

AACGCTTCACGAATTTGCGT

CTBP1-AS2

Forward primer

CACGTGTGGAGCCCTTGTAG

Reverse primer

ACCAACCACATCGTCCCTTC

ZNF217

Forward primer

ACCAACCACATCGTCCCTTC

Reverse primer

TCTCTTTTGTGCCATGCTGTT

GAPDH

Forward primer

GGAGCGAGATCCCTCCAAAAT

Reverse primer

GGCTGTTGTCATACTTCTCATGG
