# Supplementary figures and images for: Long non-coding RNA CTBP1-AS2 enhances cervical cancer progression via up-regulation of ZNF217 through sponging miR-3163
Source: Cancer Cell Int. 2020 Jul 28;20:343. doi: 10.1186/s12935-020-01430-5 (PMC7388209; doi:10.1186/s12935-020-01430-5)

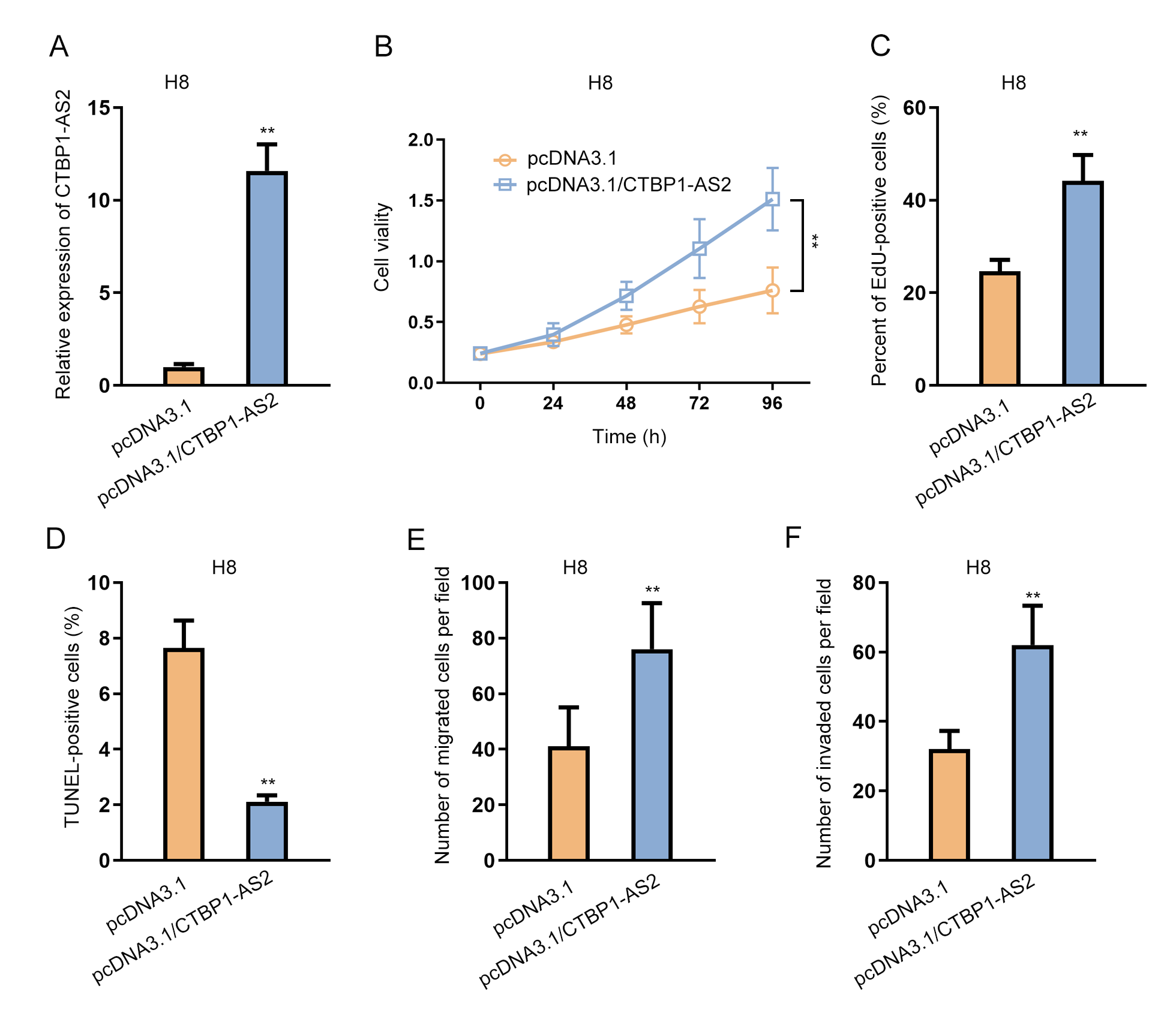

Supplement: Supplementary file 2 — Additional file 2: Figure S1. Upregulation of CTBP1-AS2 facilitates the malignant processes of H8 cell. A. CTBP1-AS2 was overexpressed in H8 cells by transfecting with CTBP1-AS2 expression vector. B-C. Cell proliferation was measured by CCK-8 and EdU assays. D. TUNEL assay was applied to analyze the apoptosis of H8 cells under the overexpression of CTBP1-AS2. E-F. The migration and invasion were detected in H8 cells after overexpression of CTBP1-AS2 by transwell assays. **P < 0.01. [file 12935_2020_1430_MOESM2_ESM.tif]

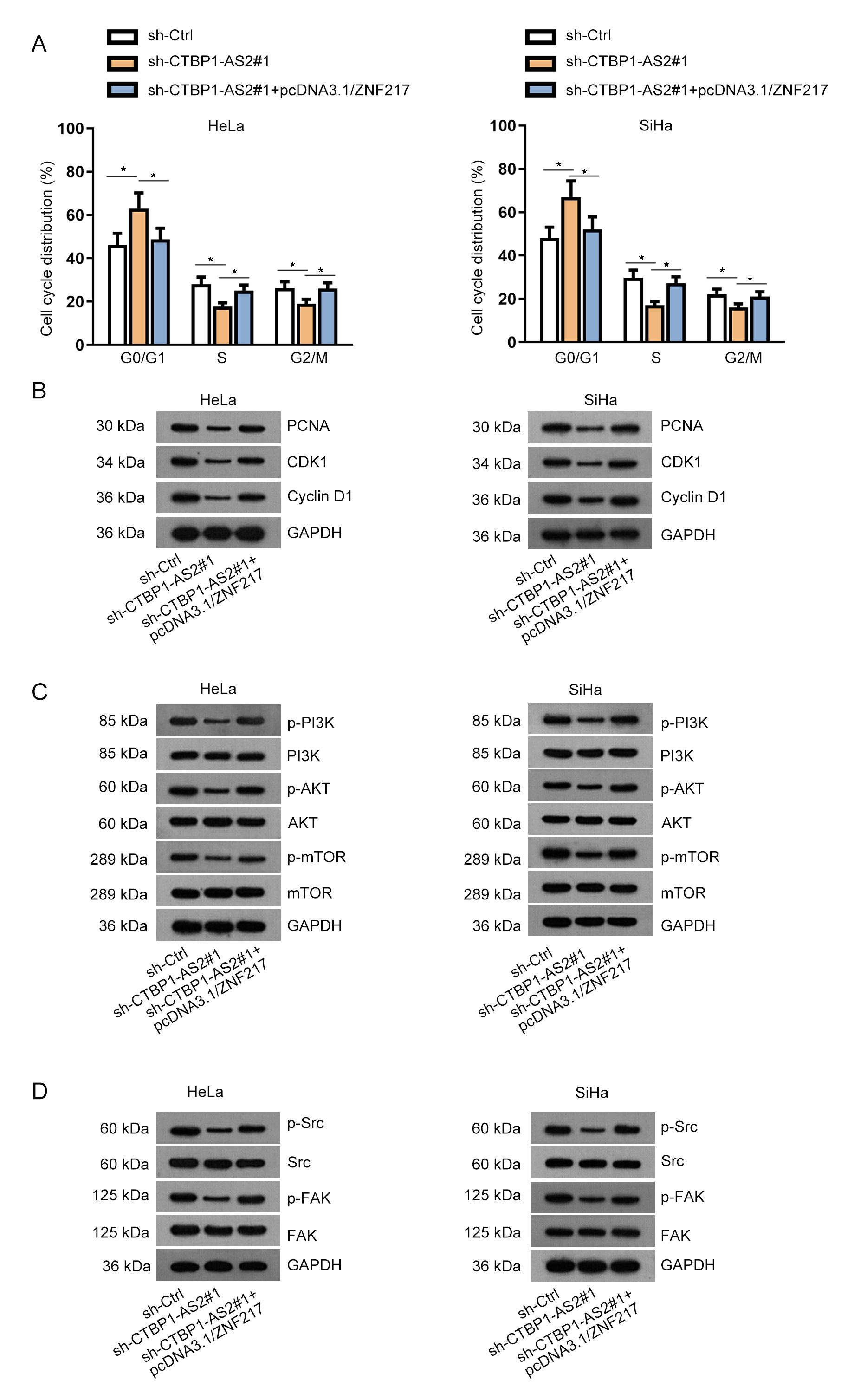

Supplement: Supplementary file 3 — Additional file 3: Figure S2. CTBP1-AS2 activates PI3K/AKT and Src/FAK signaling pathways in CC cells by upregulating ZNF217. A. Cell cycle distribution was examined in two CC cells transfected with sh-Ctrl, sh-CTBP1-AS2#1, or co-transfected with sh-CTBP1-AS2#1 and pcDNA3.1/ZNF217. B. The levels of proliferation maker PCNA and cell cycle-related proteins were evaluated by western blot analysis. C-D. Western blot analysis of PI3K/AKT pathway related proteins (p-PI3K, PI3K, p-AKT, AKT, p-mTOR, mTOR, p-Src, Src, p-FAK, FAK) and Src/FAK pathway related proteins. *P < 0.05. [file 12935_2020_1430_MOESM3_ESM.tif]

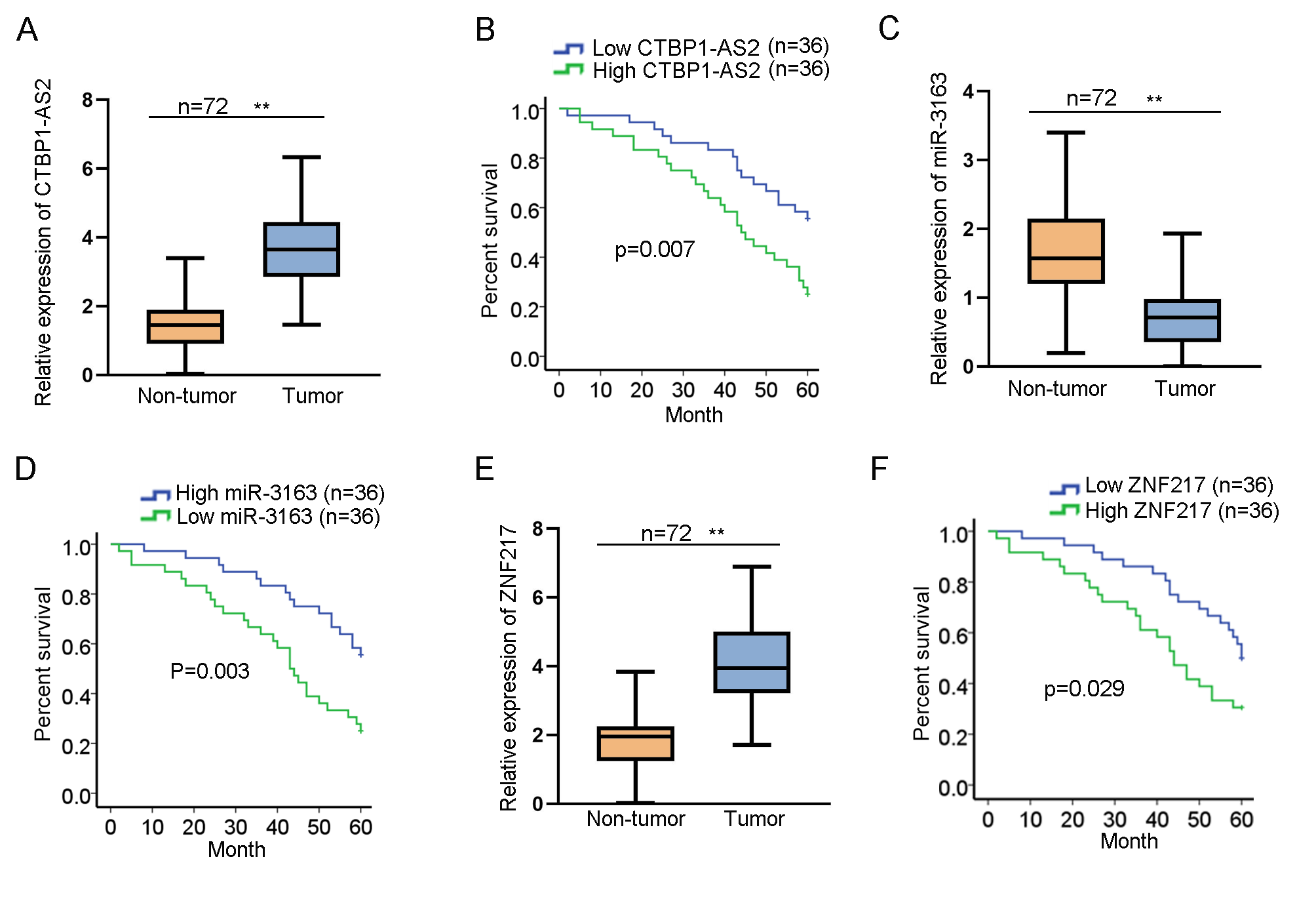

Supplement: Supplementary file 4 — Additional file 4: Figure S3. Clinical relevance of CTBP1-AS2/miR-3163/ZNF217 axis with CC patients. A. Expression of CTBP1-AS2 in CC tissues and normal tissues was revealed by qRT-PCR assay. B. Kaplan–Meier survival analysis of high expression or low expression of CTBP1-AS2 in CC patients. C. Expression of miR-3163 in CC tissues and normal tissues was revealed by qRT-PCR assay. D. Kaplan–Meier survival analysis of high expression or low expression of miR-3163 in CC patients. E. Expression of ZNF217 in CC tissues and normal tissues was revealed by qRT-PCR assay. F. Kaplan–Meier survival analysis of high expression or low expression of ZNF217 in CC patients. **P < 0.01. [file 12935_2020_1430_MOESM4_ESM.tif]
